# Supplementary figures and images for: A SUMO E3 ligase promotes long non-coding RNA transcription to regulate small RNA-directed DNA elimination
Source: eLife. 2024 Jan 10;13:e95337. doi: 10.7554/eLife.95337 (PMC10830130; doi:10.7554/eLife.95337)

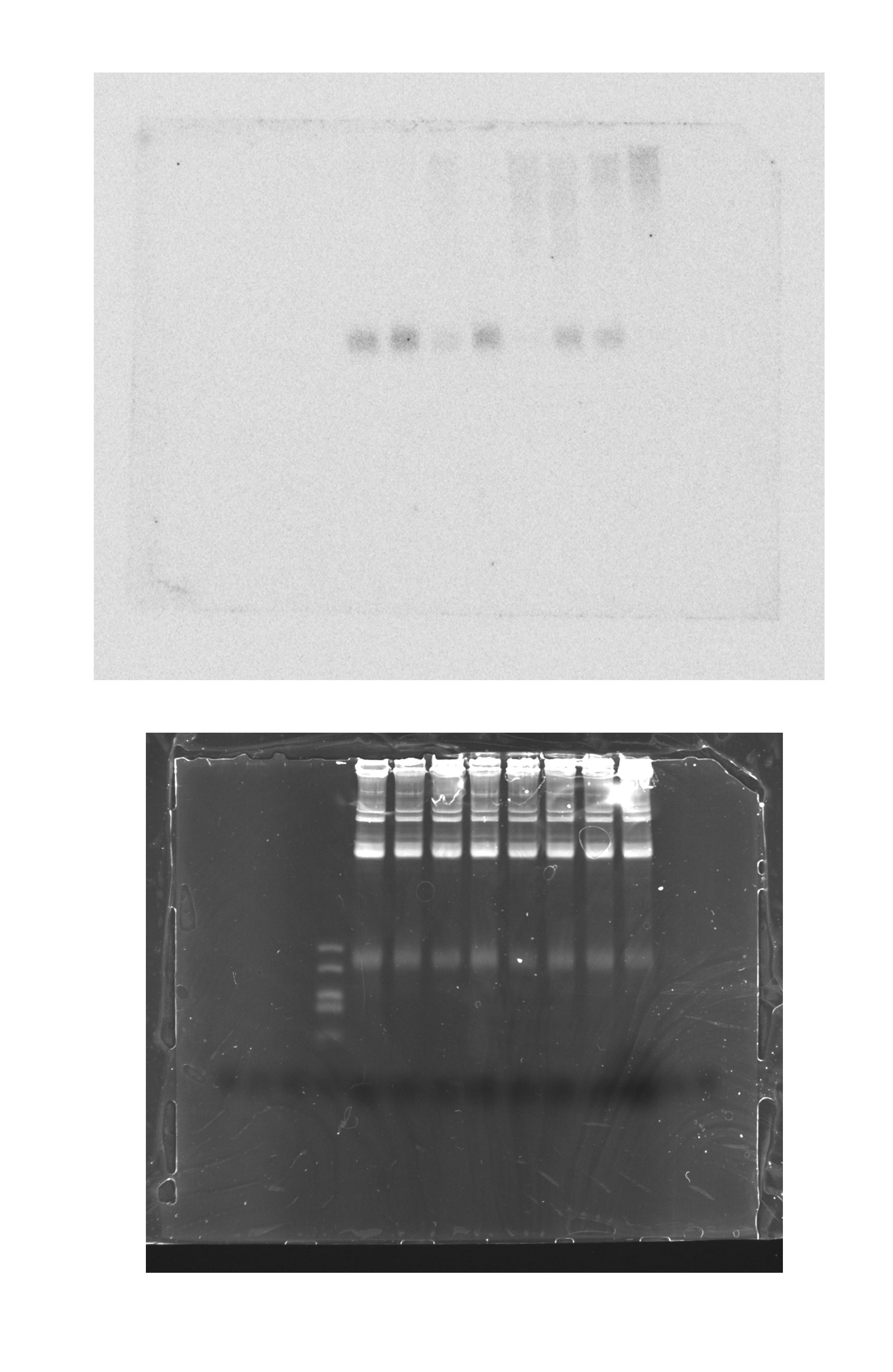

Supplement: Figure 3—source data 1. [file elife-95337-fig3-data1.zip › Figure_3A_Original.jpg]

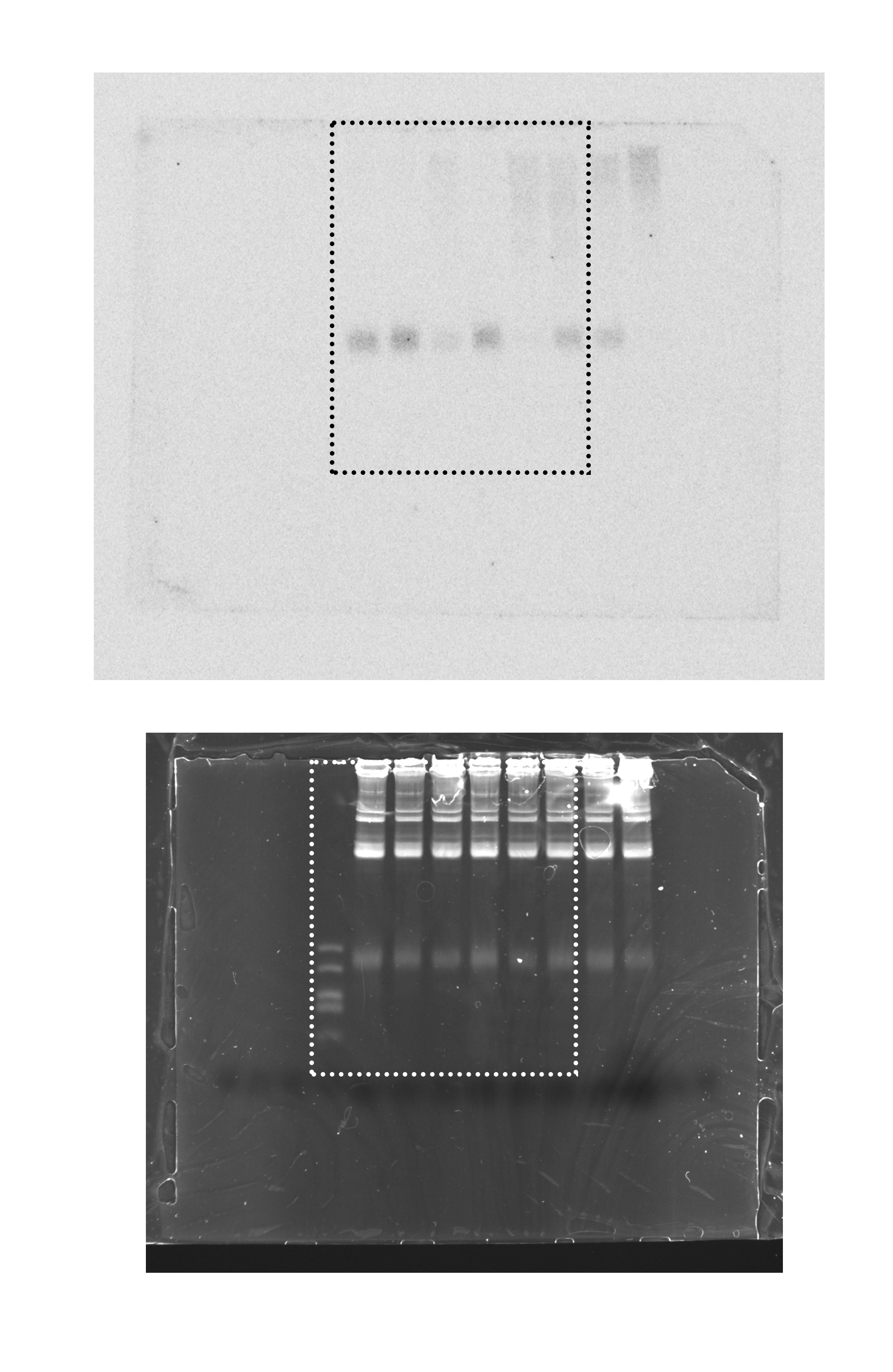

Supplement: Figure 3—source data 1. [file elife-95337-fig3-data1.zip › Figure_3A_Original-marked.jpg]

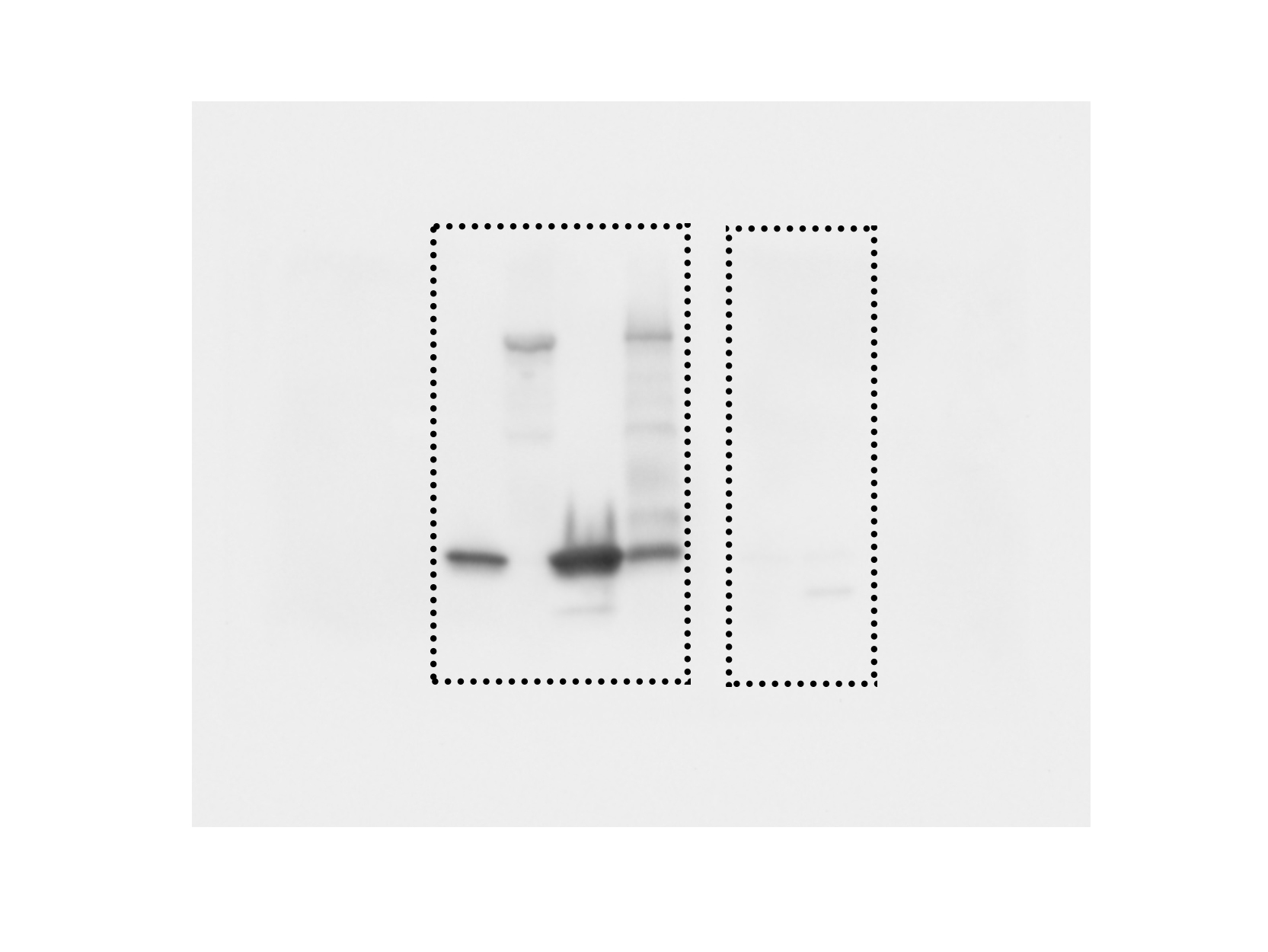

Supplement: Figure 4—source data 1. [file elife-95337-fig4-data1.zip › Figure 4-source data 1/Figure_4B_Original-marked.jpg]

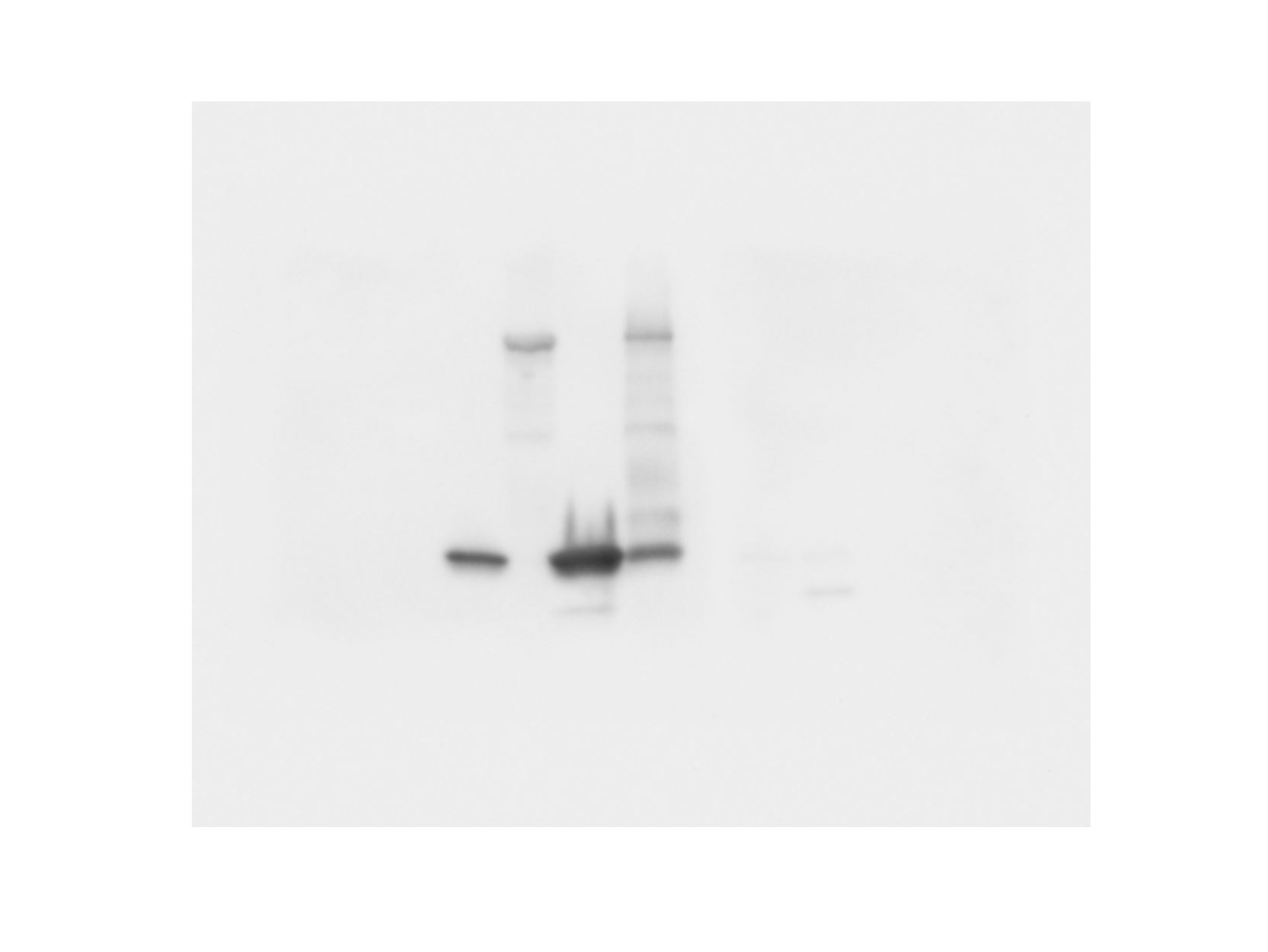

Supplement: Figure 4—source data 1. [file elife-95337-fig4-data1.zip › Figure 4-source data 1/Figure_4B_Original.jpg]

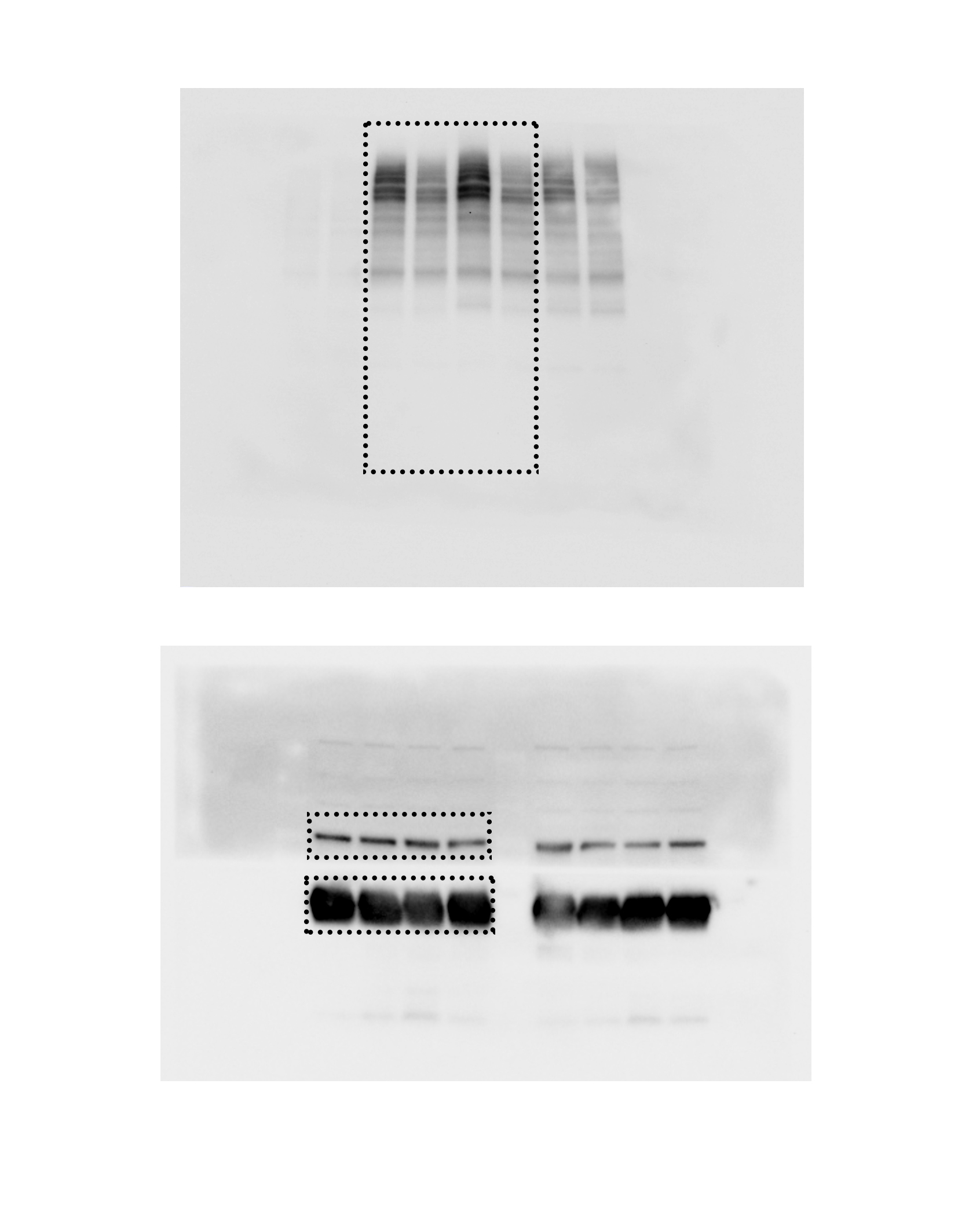

Supplement: Figure 4—source data 2. [file elife-95337-fig4-data2.zip › Figure 4-source data 2/Figure_4C_Original-marked.jpg]

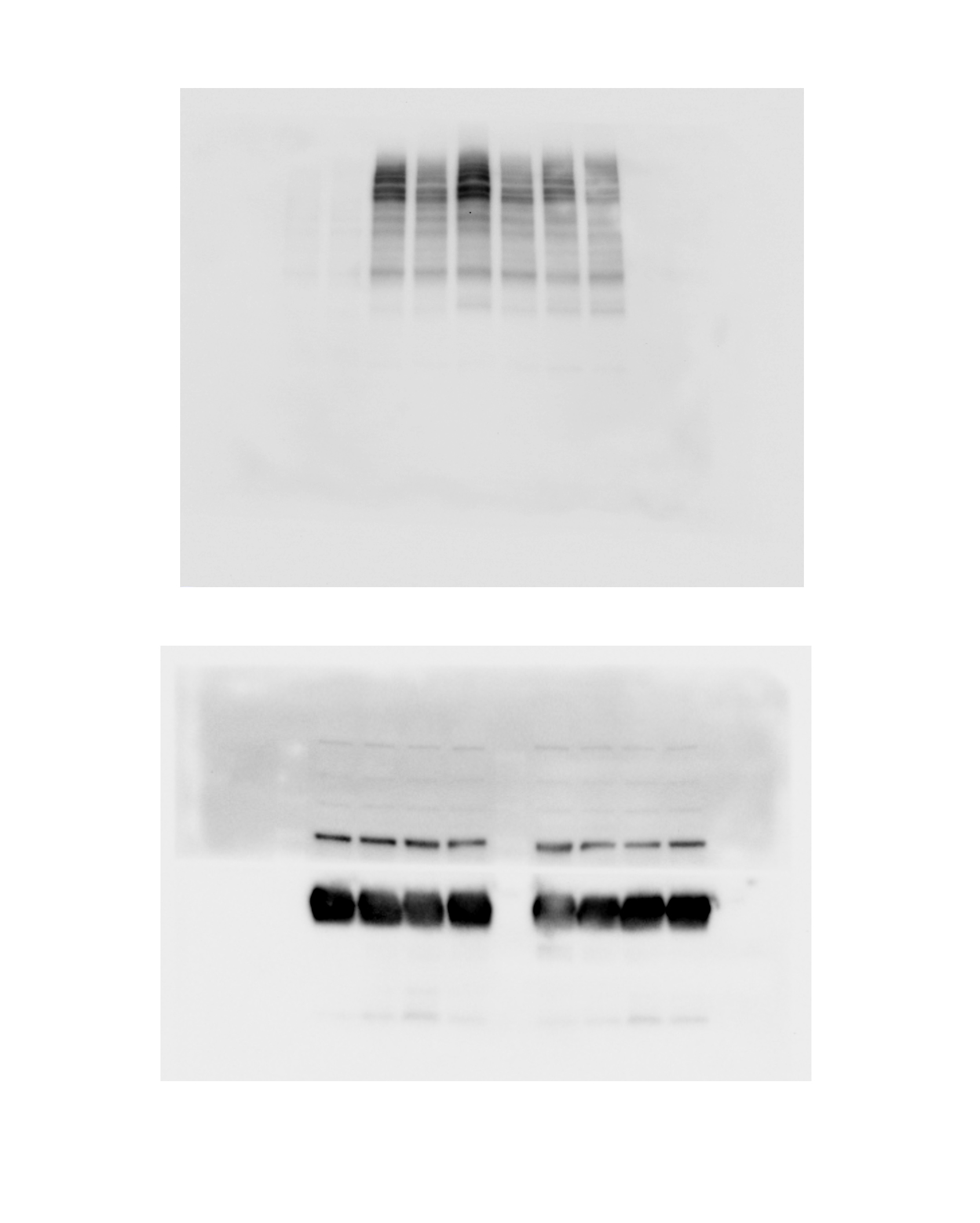

Supplement: Figure 4—source data 2. [file elife-95337-fig4-data2.zip › Figure 4-source data 2/Figure_4C_Original.jpg]

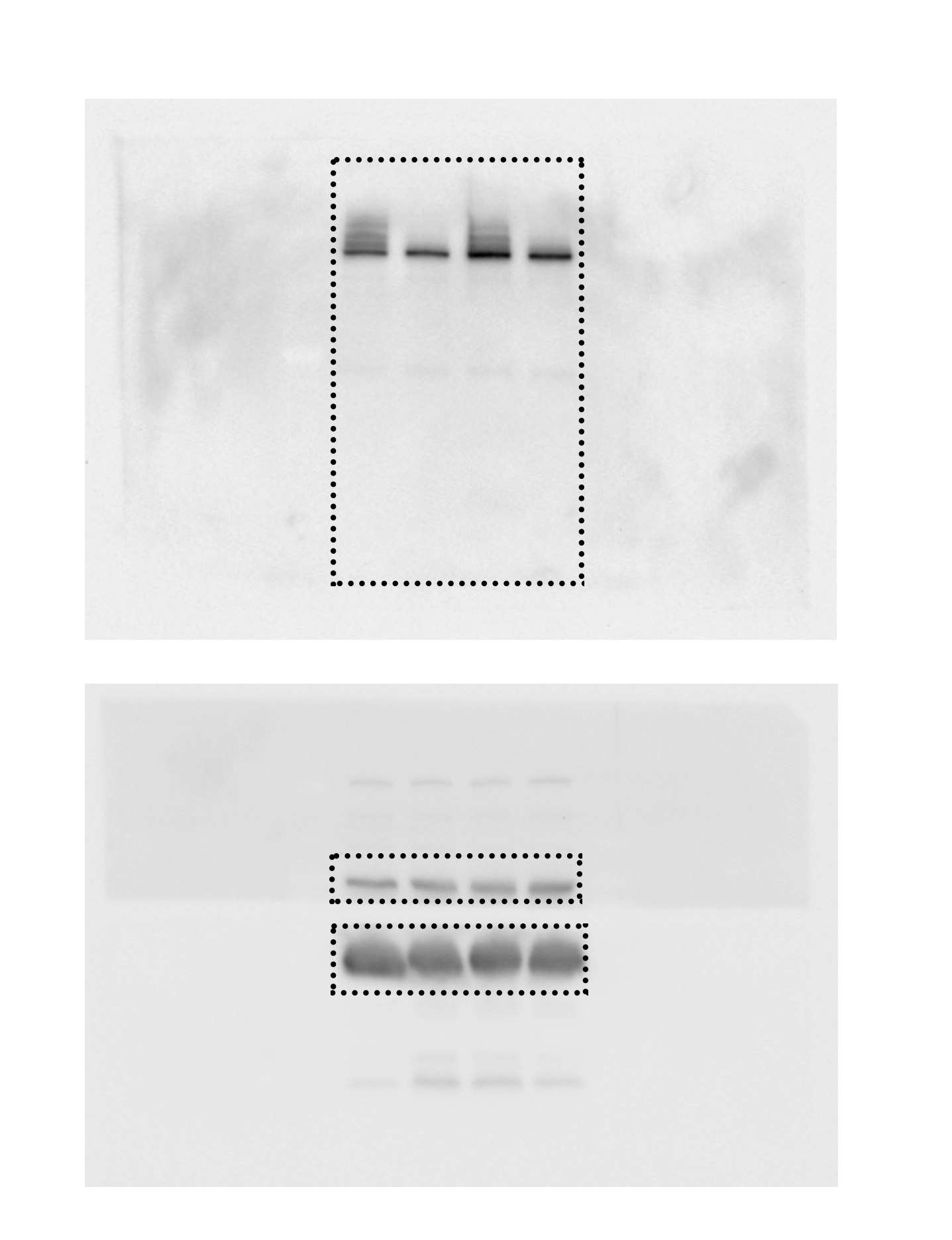

Supplement: Figure 5—source data 1. [file elife-95337-fig5-data1.zip › Figure 5-source data 1/Figure_5B_Original-marked.jpg]

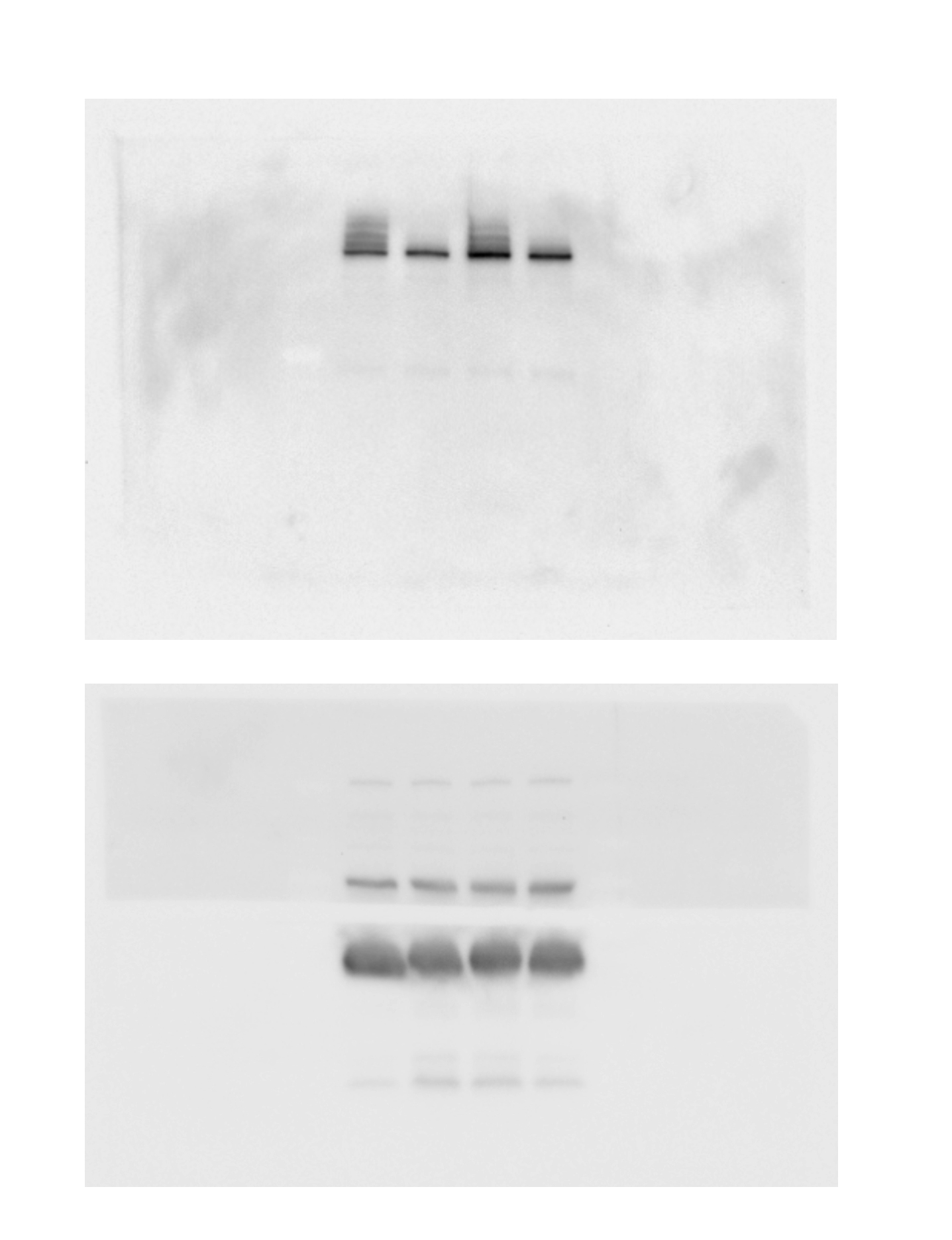

Supplement: Figure 5—source data 1. [file elife-95337-fig5-data1.zip › Figure 5-source data 1/Figure_5B_Original.jpg]

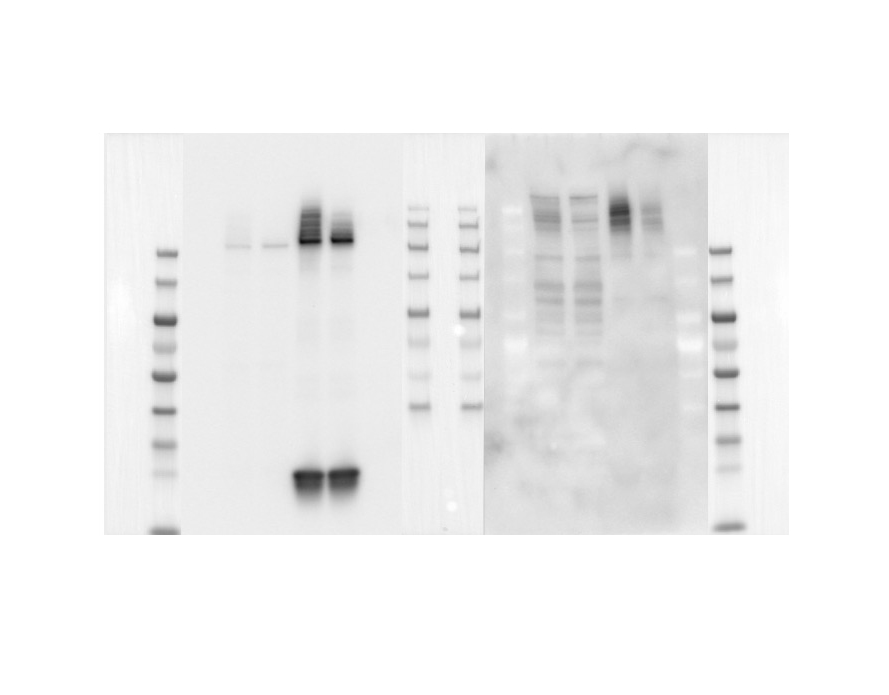

Supplement: Figure 5—source data 2. [file elife-95337-fig5-data2.zip › Figure 5-source data 2/Figure_5C_Original.jpg]

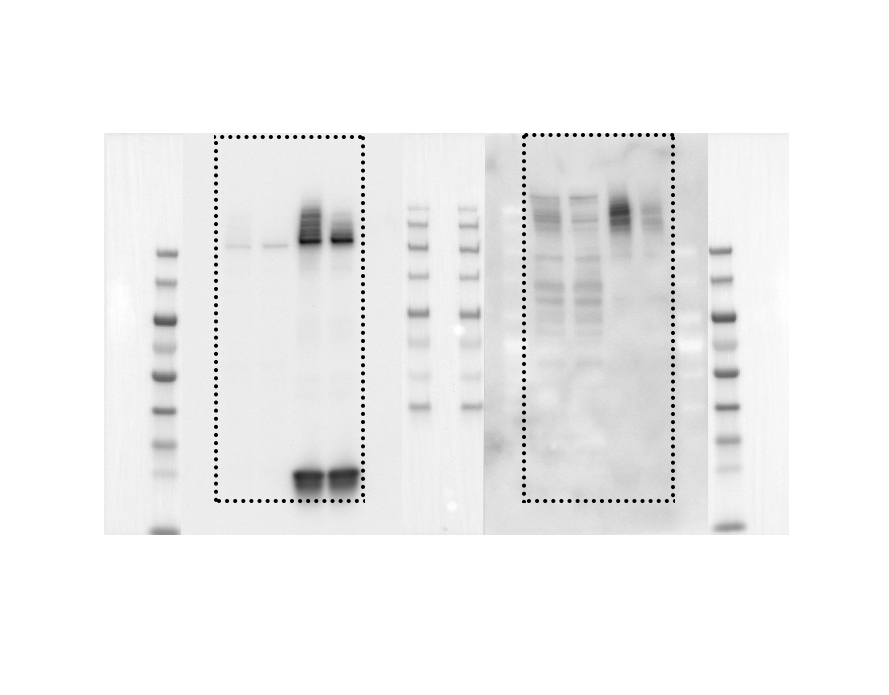

Supplement: Figure 5—source data 2. [file elife-95337-fig5-data2.zip › Figure 5-source data 2/Figure_5C_Original-marked.jpg]

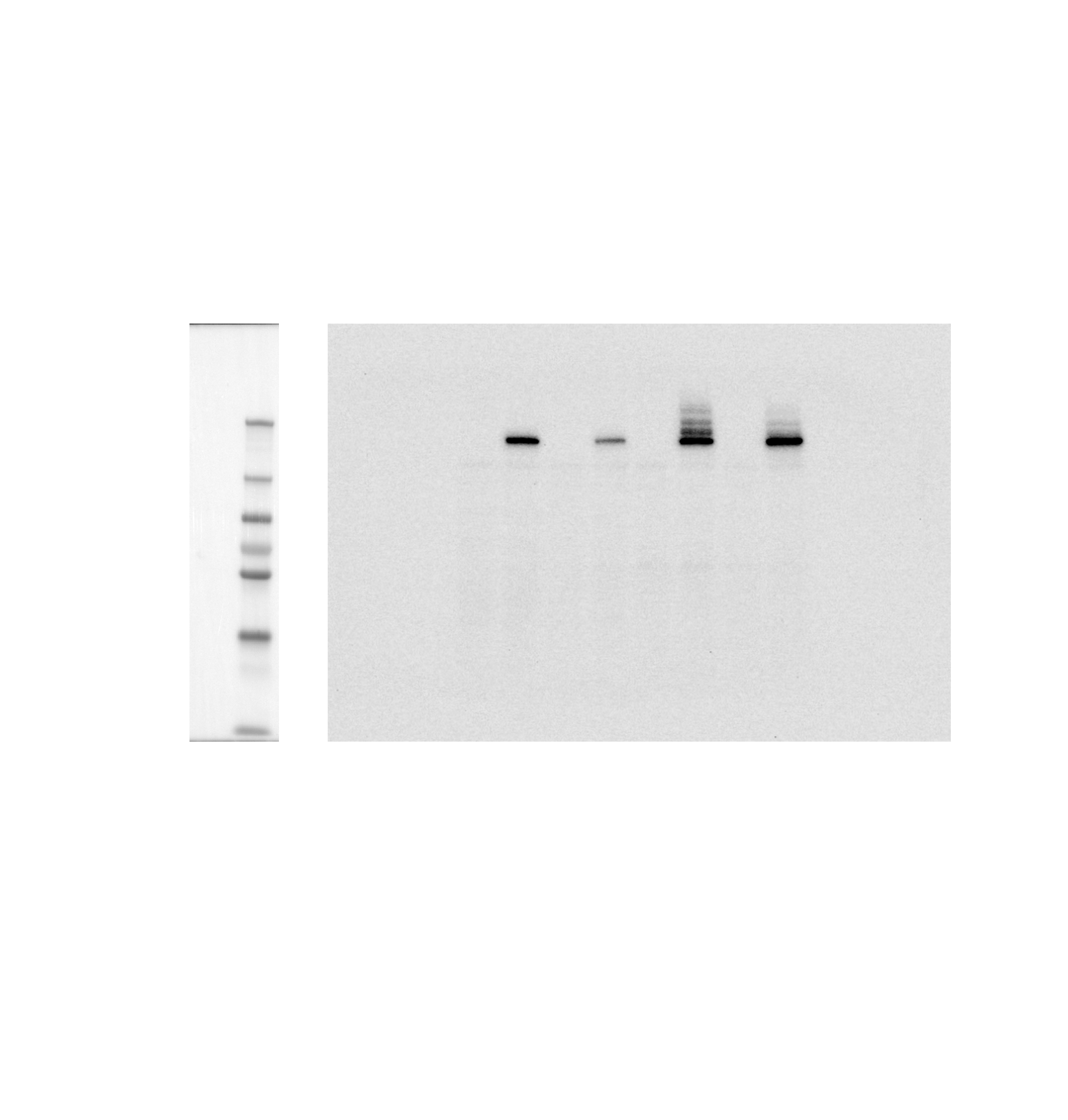

Supplement: Figure 5—figure supplement 1—source data 1. [file elife-95337-fig5-figsupp1-data1.zip › Figure 5-figure supplement 1-source data 1/Figure_5-figure supplement_1_Original.jpg]

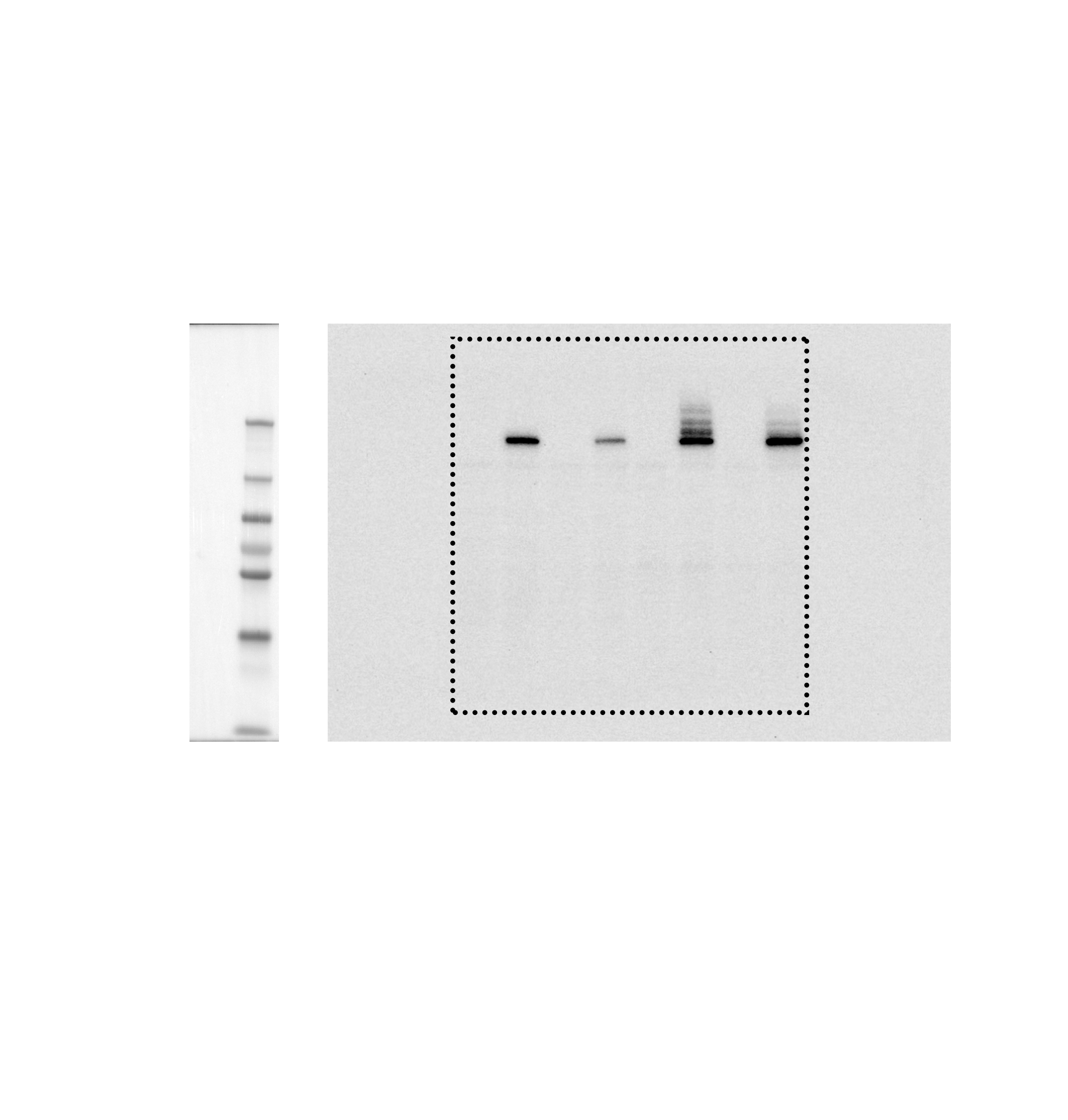

Supplement: Figure 5—figure supplement 1—source data 1. [file elife-95337-fig5-figsupp1-data1.zip › Figure 5-figure supplement 1-source data 1/Figure_5-figure supplement_1_Original-marked.jpg]

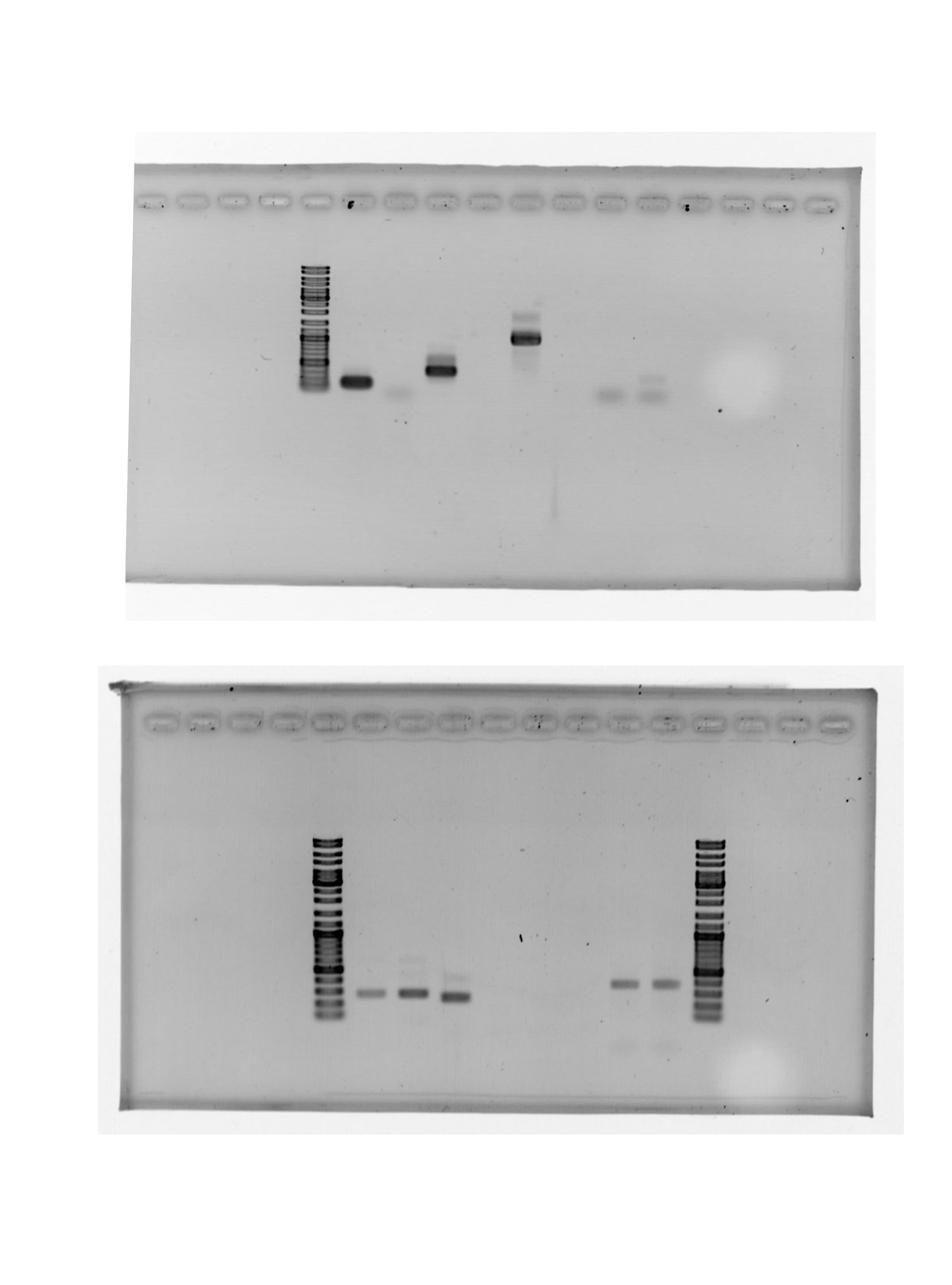

Supplement: Figure 6—source data 1. [file elife-95337-fig6-data1.zip › Figure 6-source data 1/Figure_6A_Original.jpg]

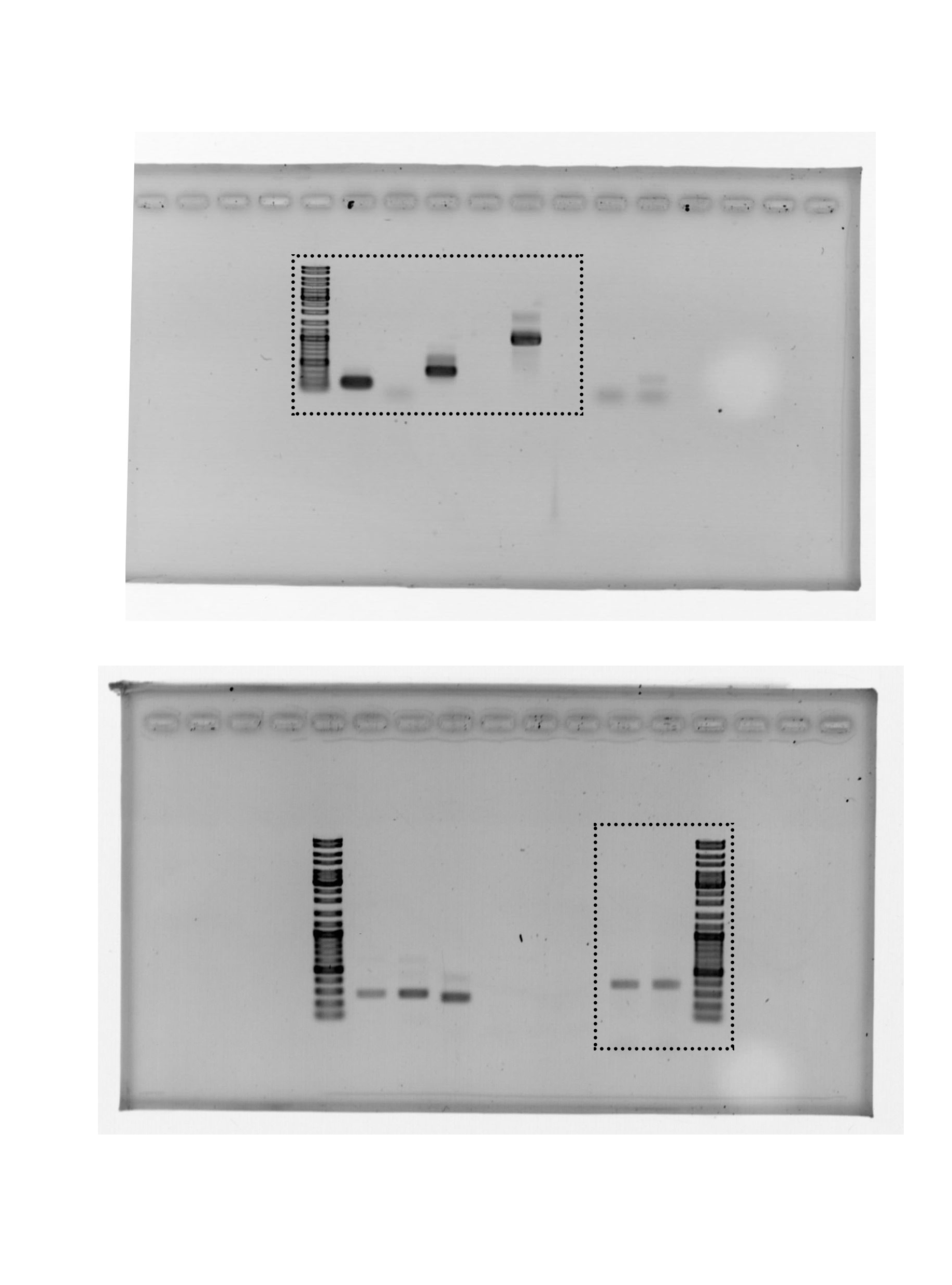

Supplement: Figure 6—source data 1. [file elife-95337-fig6-data1.zip › Figure 6-source data 1/Figure_6A_Original-marked.jpg]

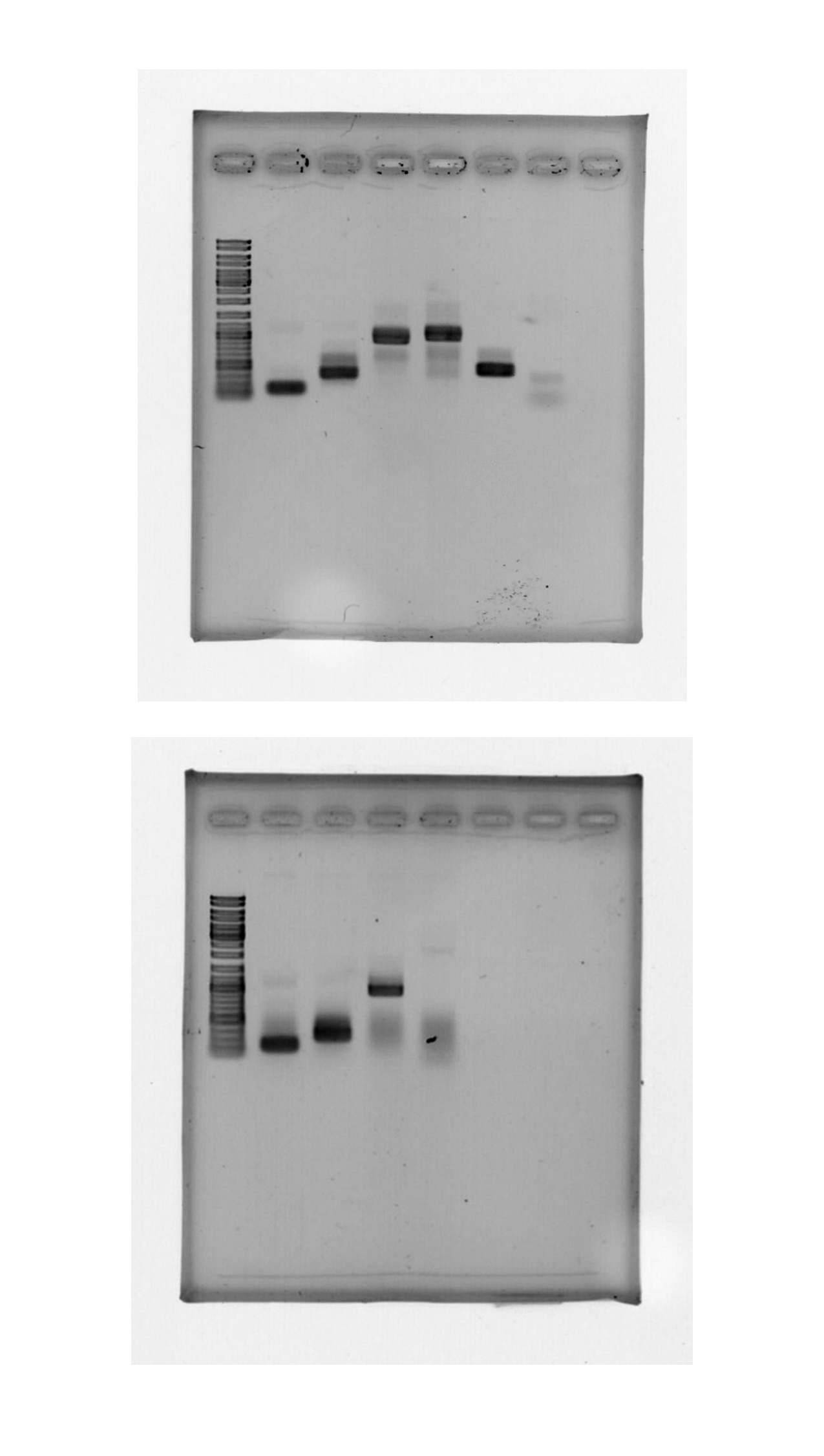

Supplement: Figure 6—figure supplement 1—source data 1. [file elife-95337-fig6-figsupp1-data1.zip › Figure 6-figure supplement 1-source data 1/Figure_6-figure supplement 1-Original.jpg]

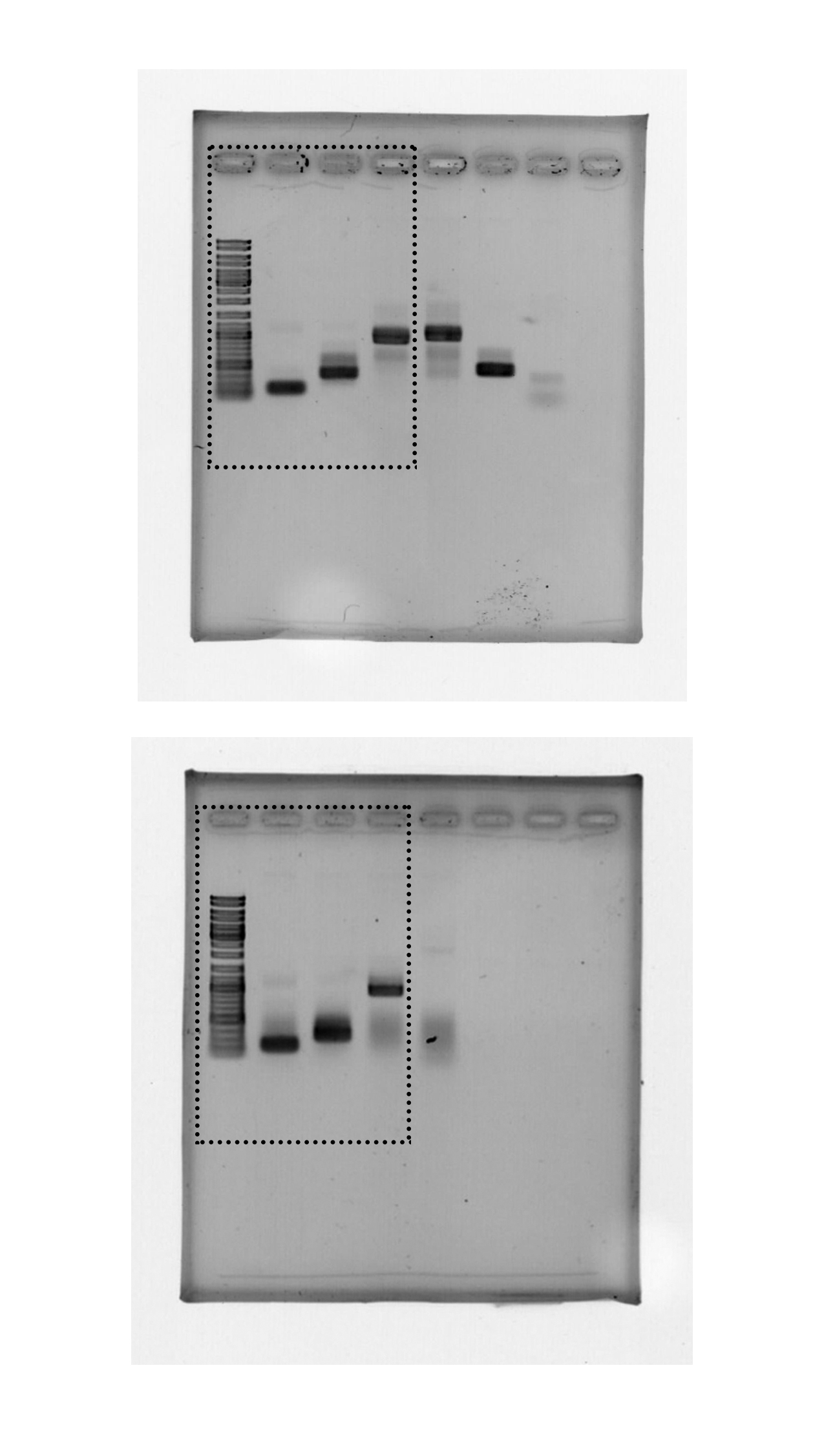

Supplement: Figure 6—figure supplement 1—source data 1. [file elife-95337-fig6-figsupp1-data1.zip › Figure 6-figure supplement 1-source data 1/Figure_6-figure supplement 1-Original-marked.jpg]

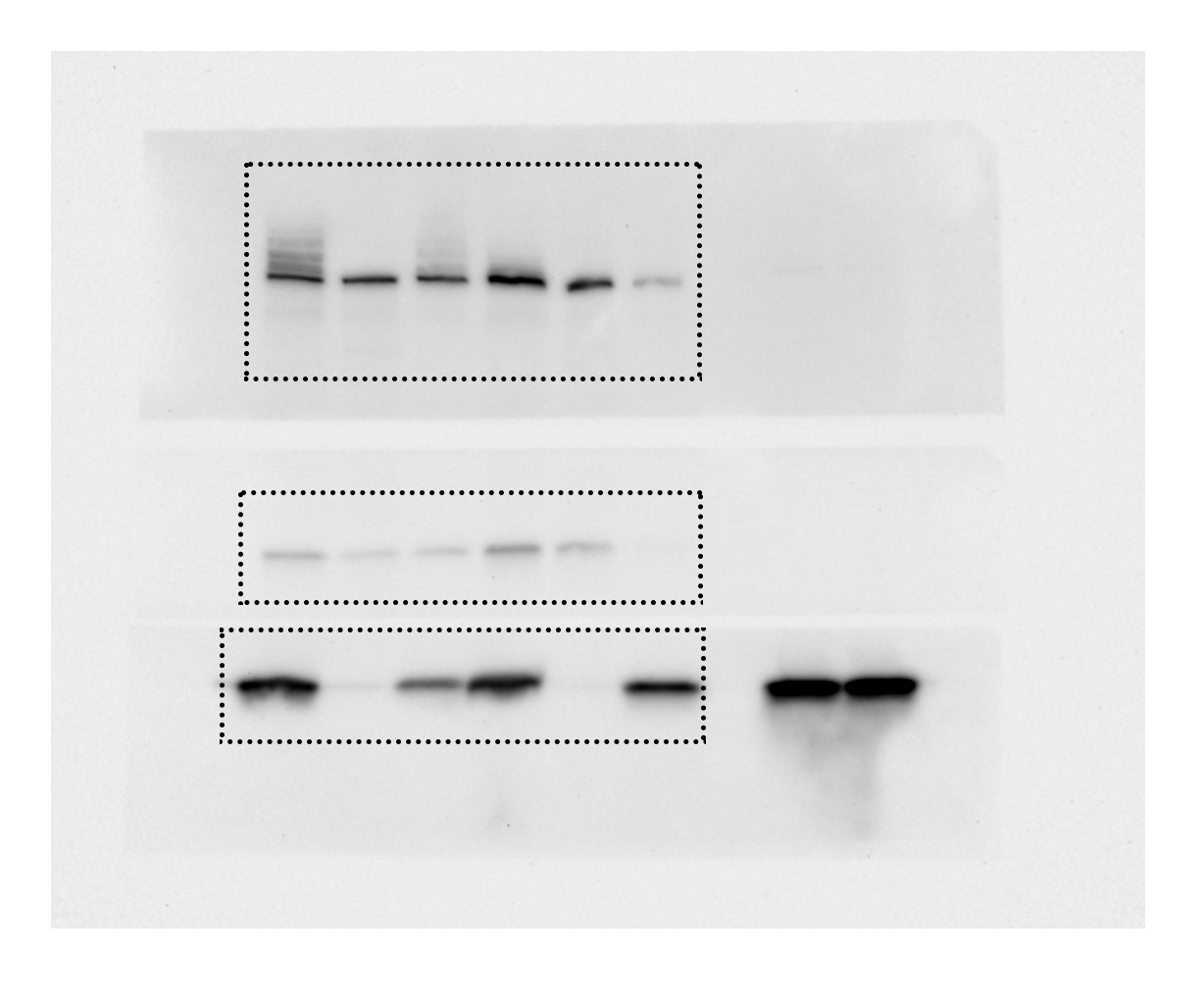

Supplement: Figure 7—source data 1. [file elife-95337-fig7-data1.zip › Figure 7-source data 1/Figure_7B-Original-marked.jpg]

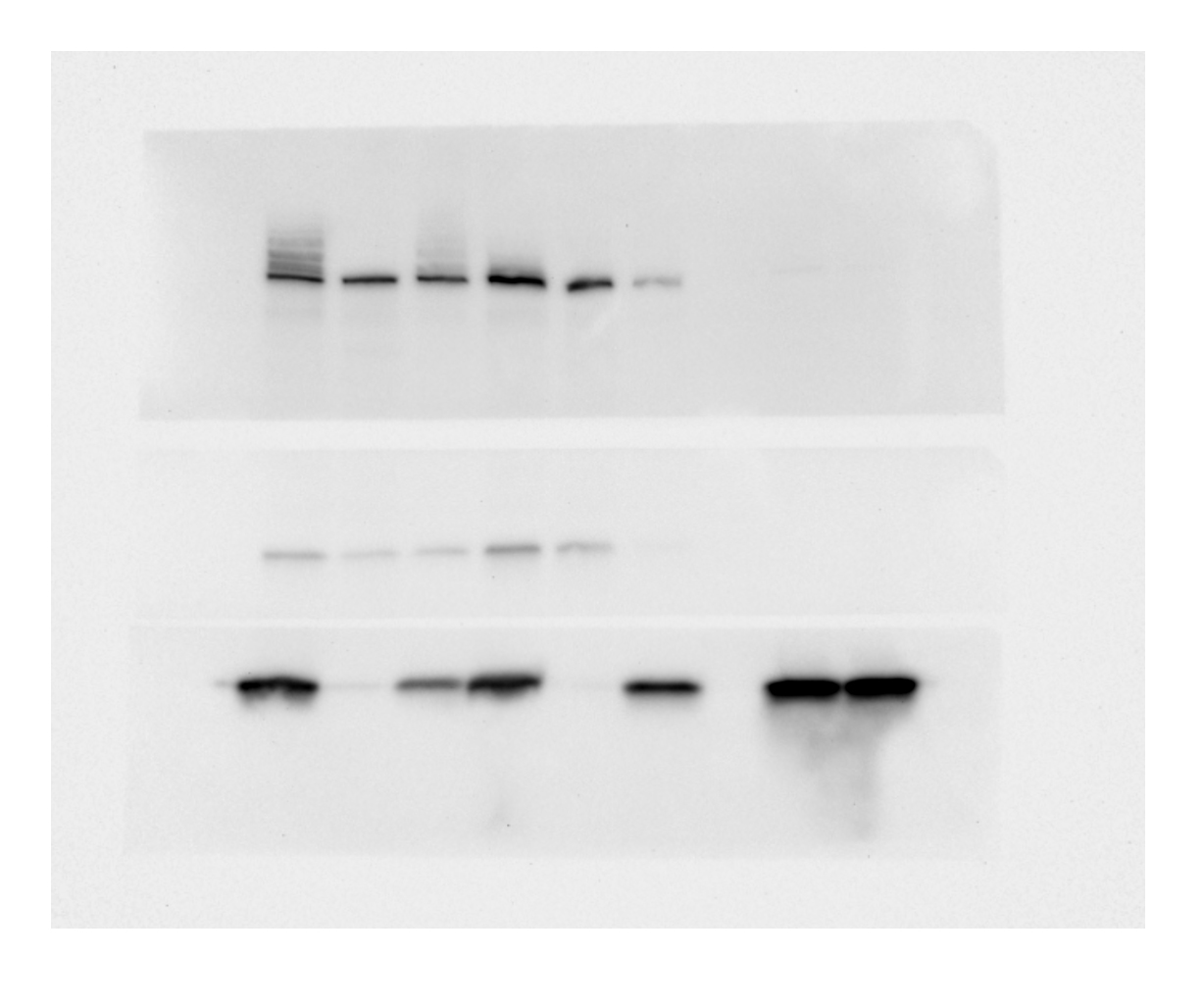

Supplement: Figure 7—source data 1. [file elife-95337-fig7-data1.zip › Figure 7-source data 1/Figure_7B-Original.jpg]

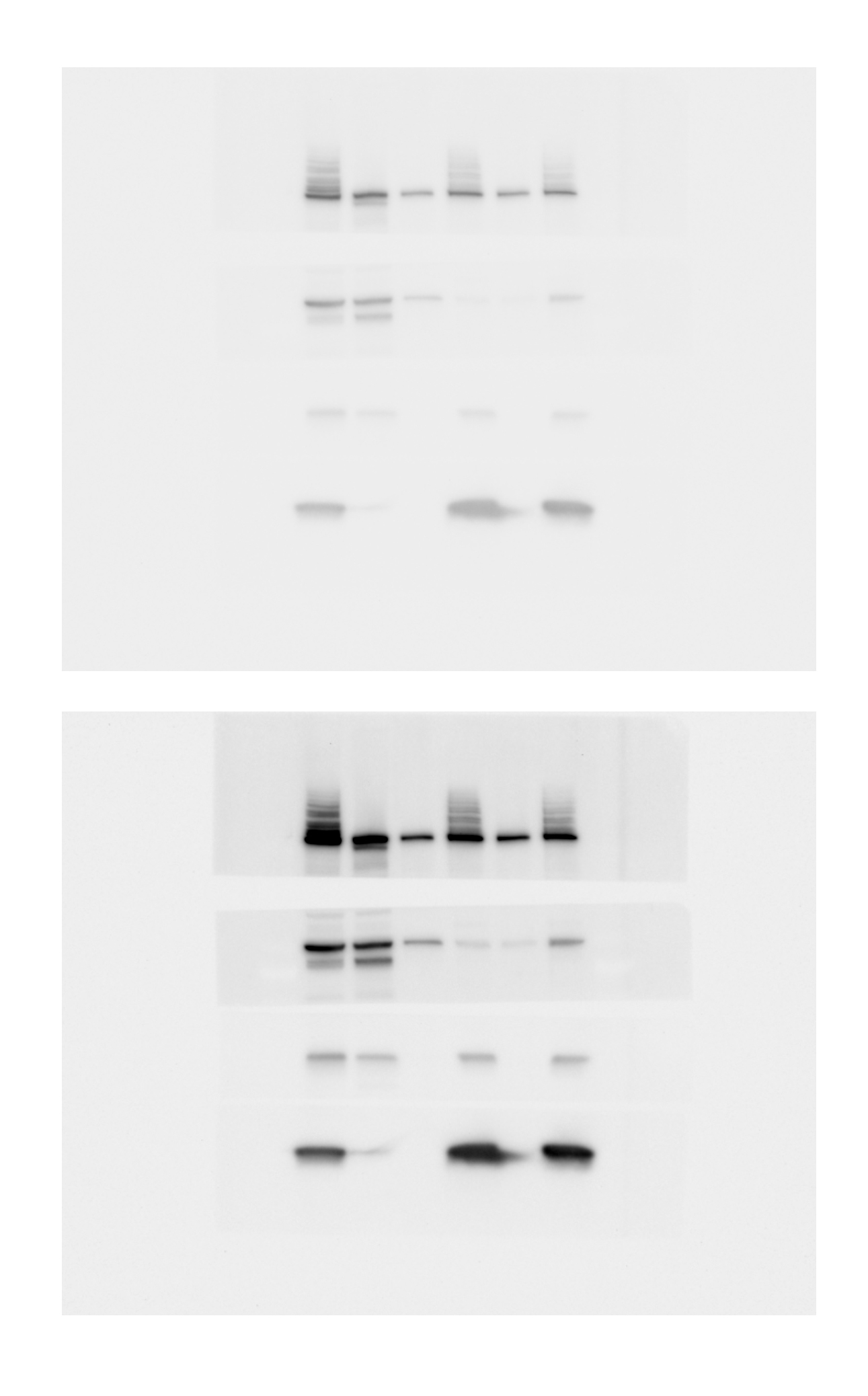

Supplement: Figure 7—source data 2. [file elife-95337-fig7-data2.zip › Figure 7-source data 2/Figure_7C-Original.jpg]

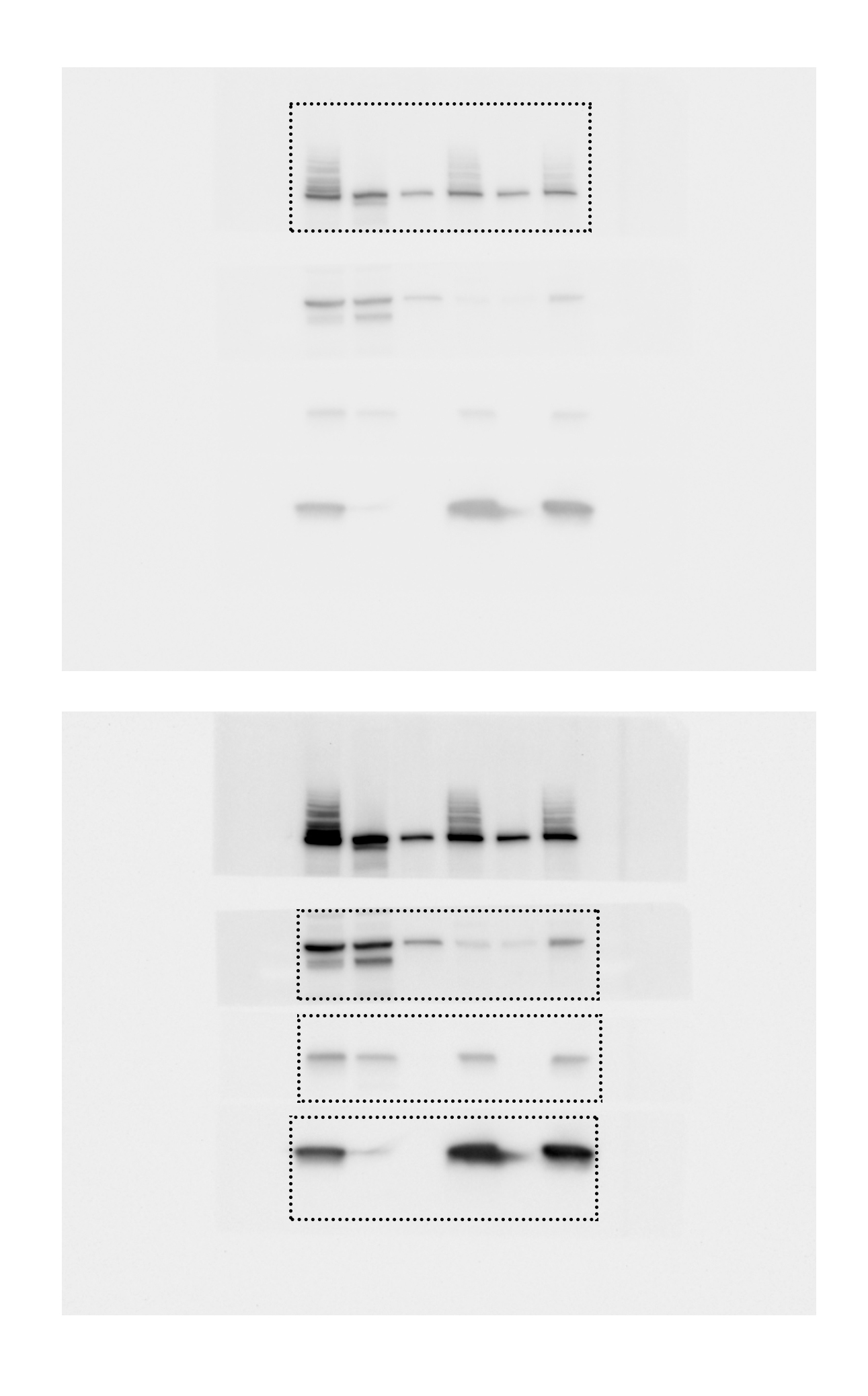

Supplement: Figure 7—source data 2. [file elife-95337-fig7-data2.zip › Figure 7-source data 2/Figure_7C-Original-marked.jpg]

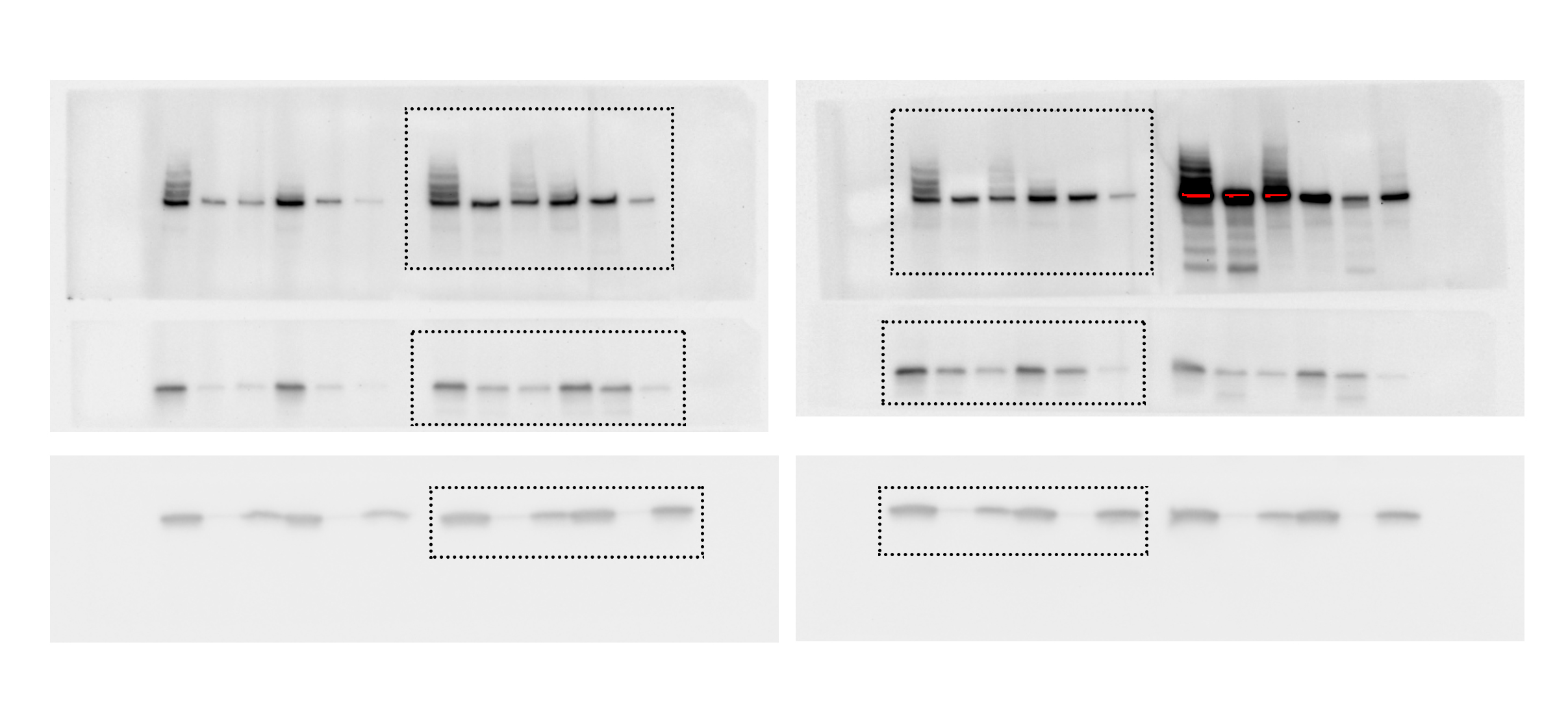

Supplement: Figure 7—figure supplement 2—source data 1. [file elife-95337-fig7-figsupp2-data1.zip › Figure 7-figure supplement 2-source data 1/Figure 7-figure supplement 2-Original-marked.jpg]

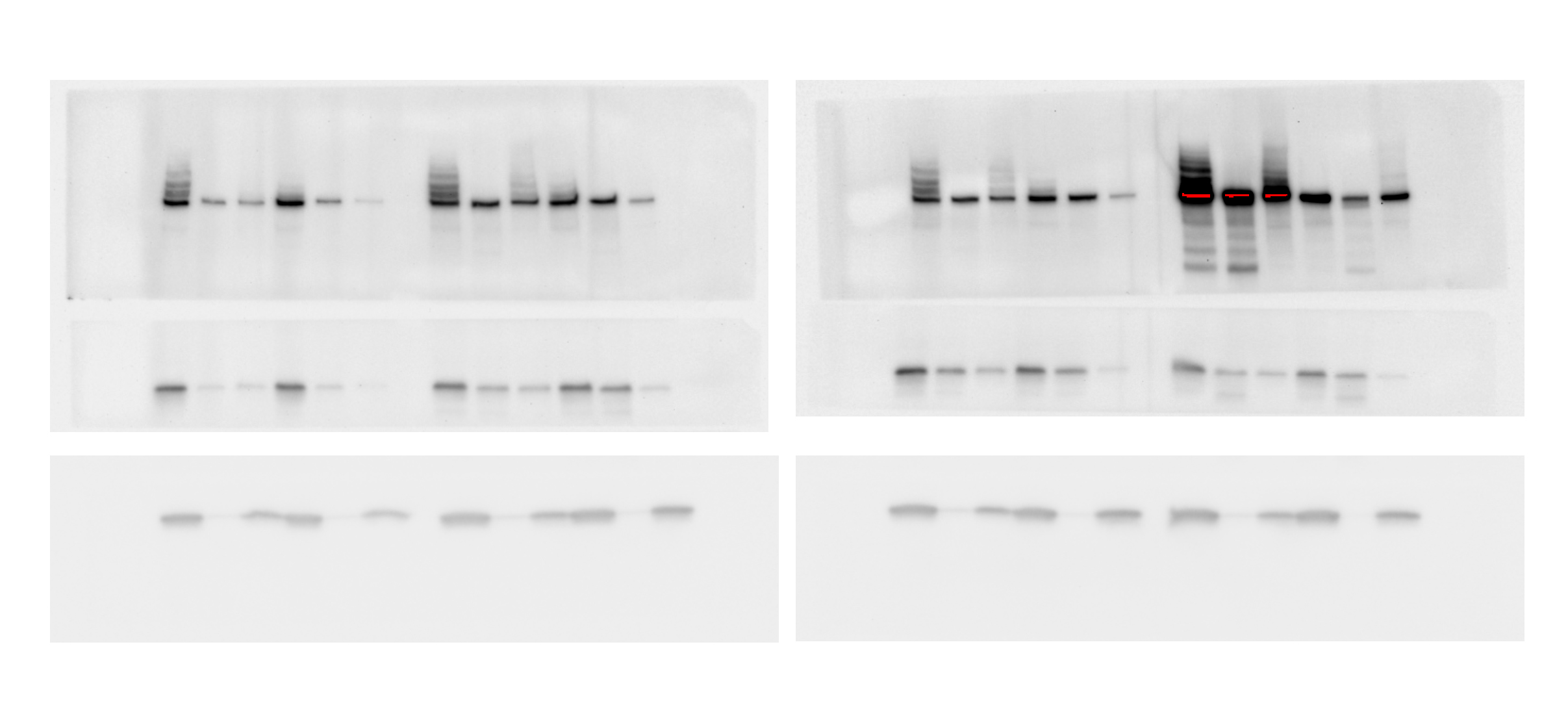

Supplement: Figure 7—figure supplement 2—source data 1. [file elife-95337-fig7-figsupp2-data1.zip › Figure 7-figure supplement 2-source data 1/Figure 7-figure supplement 2-Original.jpg]

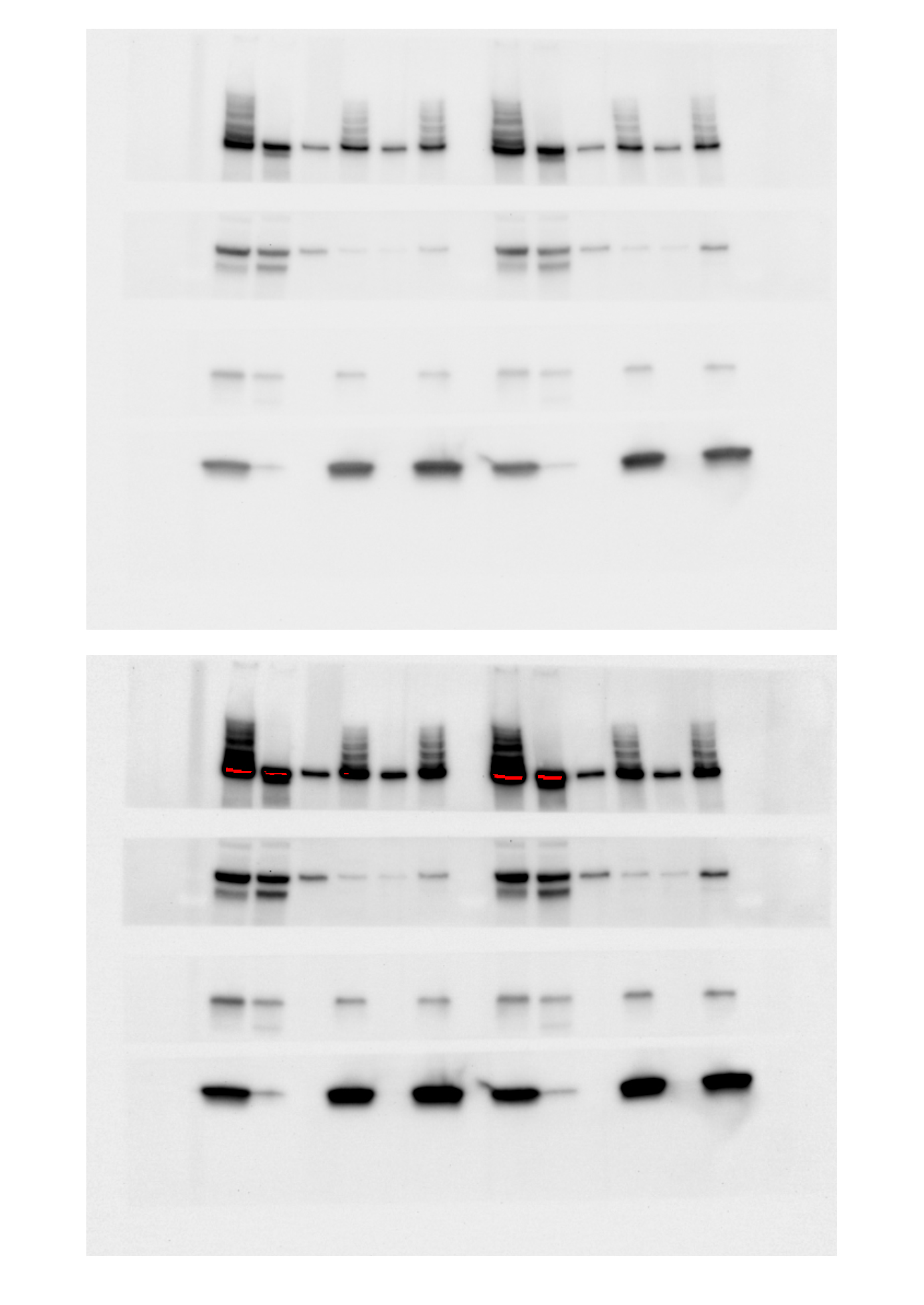

Supplement: Figure 7—figure supplement 3—source data 1. [file elife-95337-fig7-figsupp3-data1.zip › Figure 7-figure supplement 3-source data 1/Figure 7-figure supplement 3-Original.jpg]

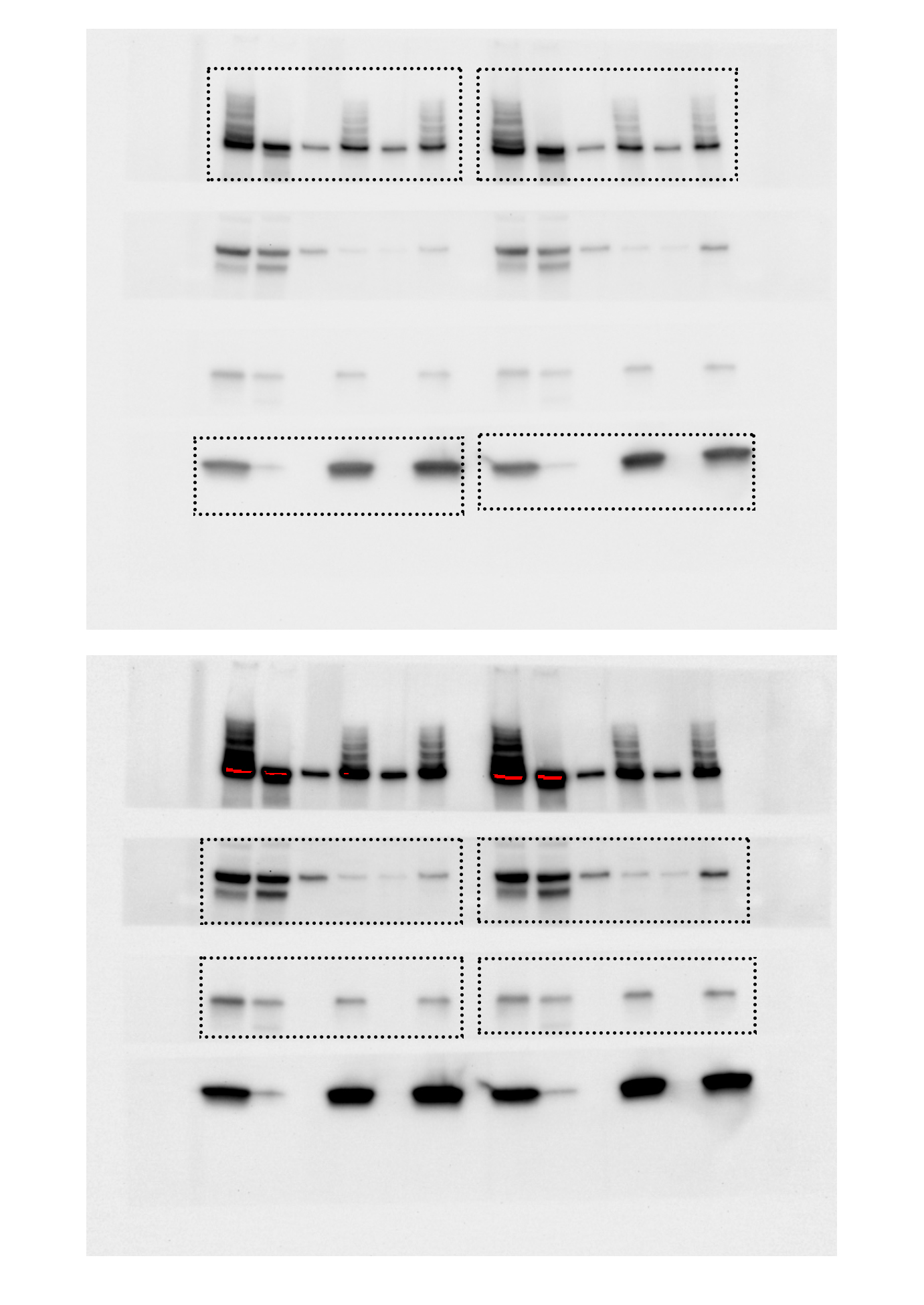

Supplement: Figure 7—figure supplement 3—source data 1. [file elife-95337-fig7-figsupp3-data1.zip › Figure 7-figure supplement 3-source data 1/Figure 7-figure supplement 3-Original-marked.jpg]

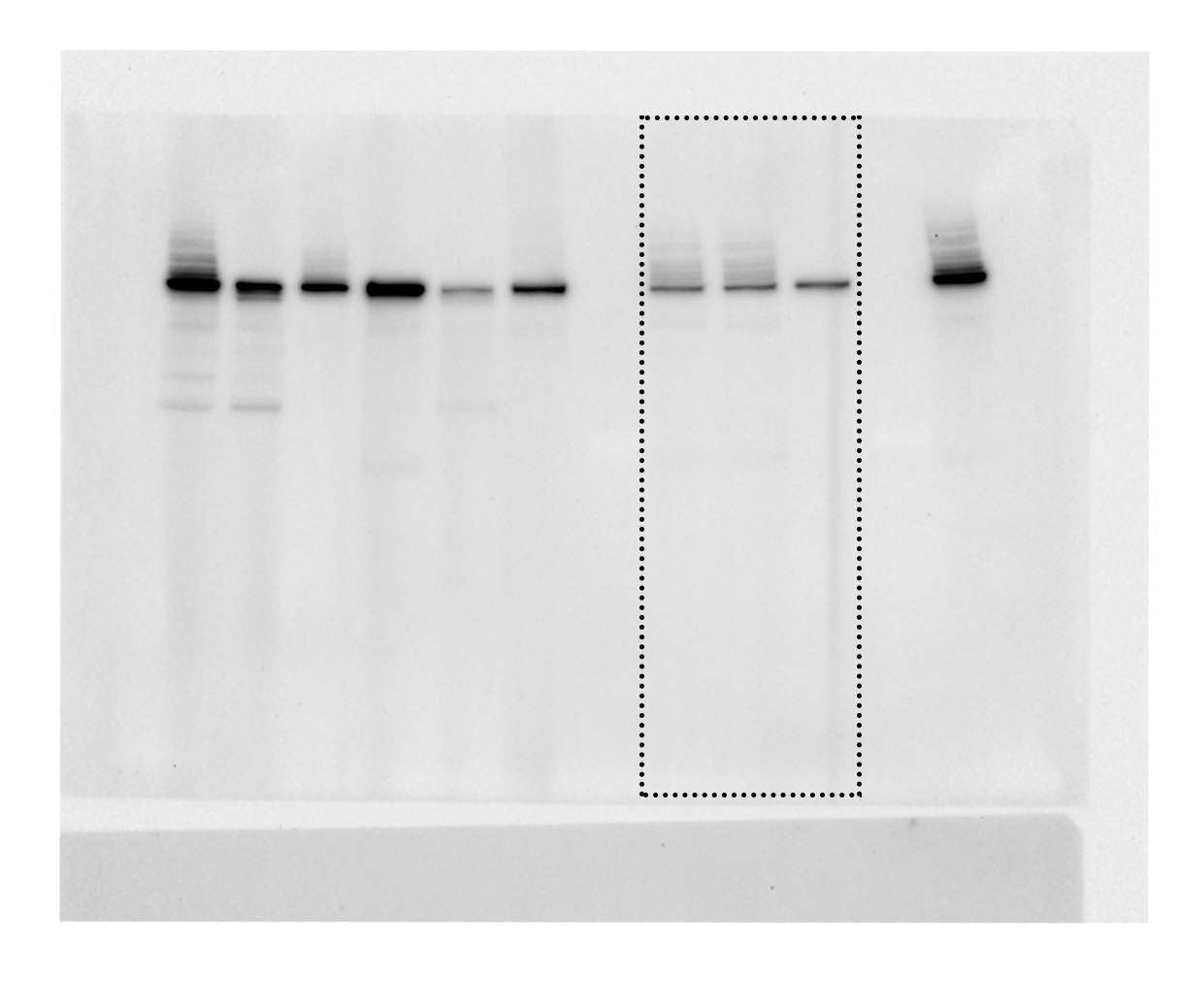

Supplement: Figure 8—source data 1. [file elife-95337-fig8-data1.zip › Figure 8-source data 1/Figure_8B-Original-marked.jpg]

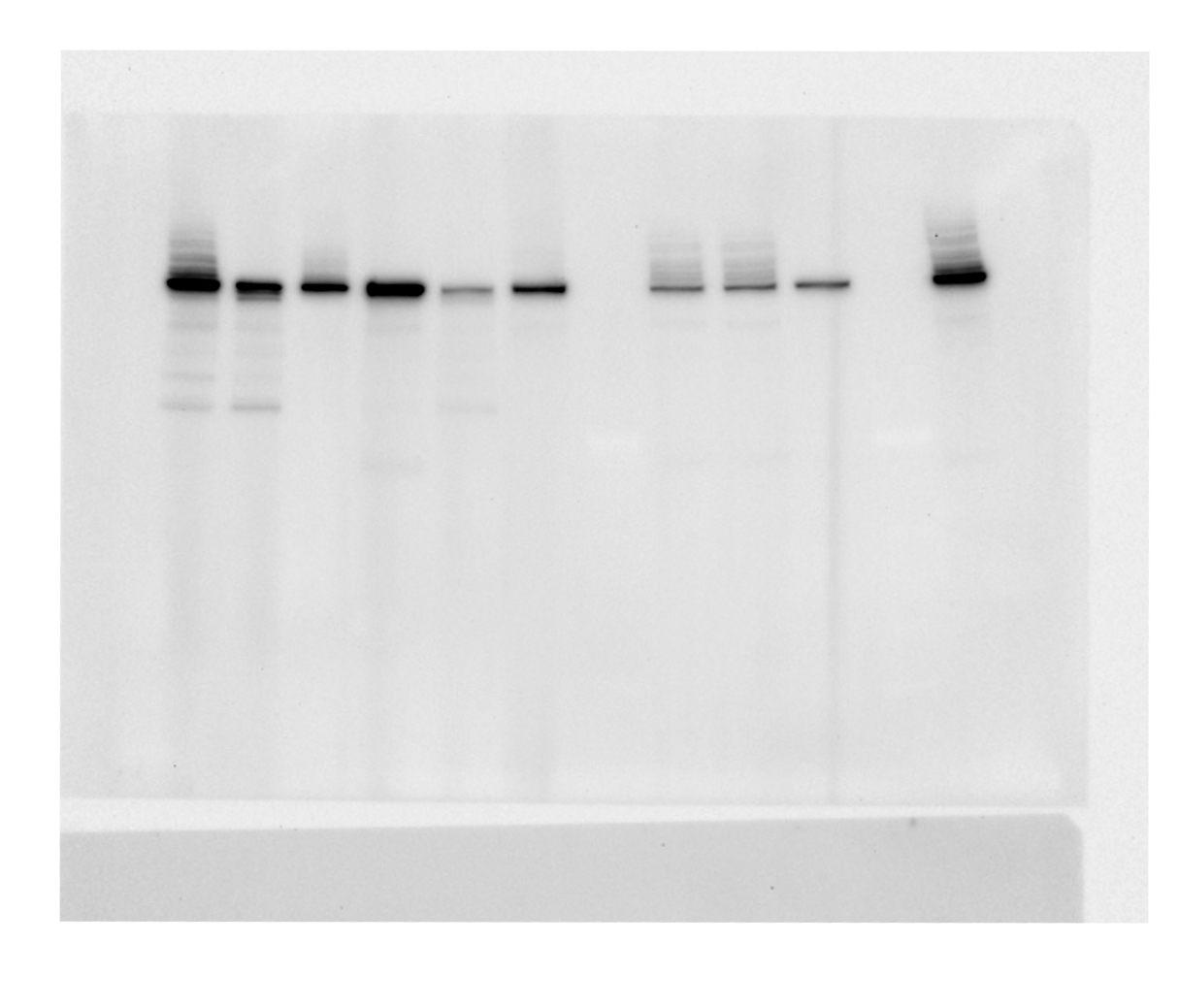

Supplement: Figure 8—source data 1. [file elife-95337-fig8-data1.zip › Figure 8-source data 1/Figure_8B-Original.jpg]

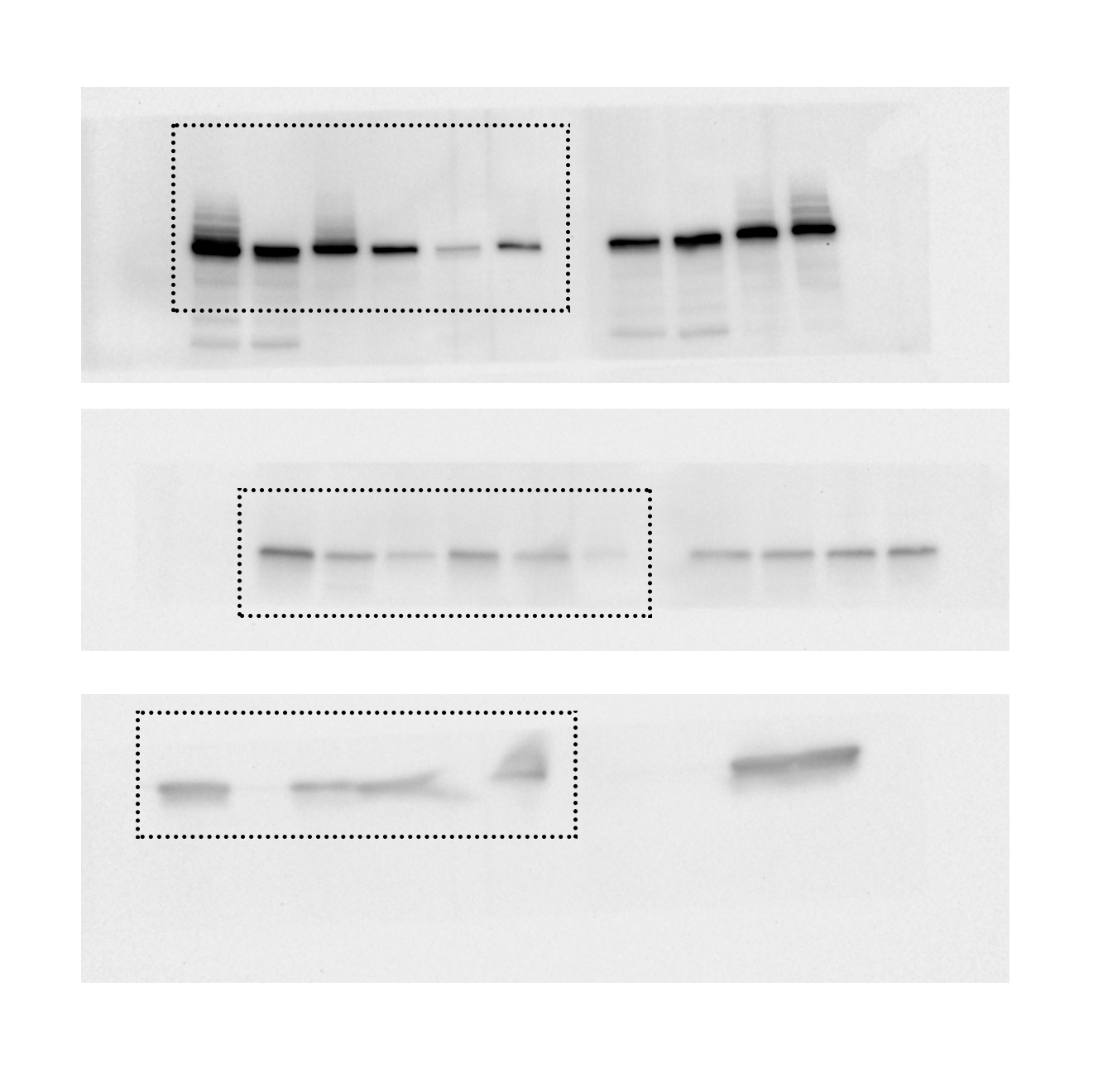

Supplement: Figure 8—source data 2. [file elife-95337-fig8-data2.zip › Figure 8-source data 2/Figure_8C-Original-marked.jpg]

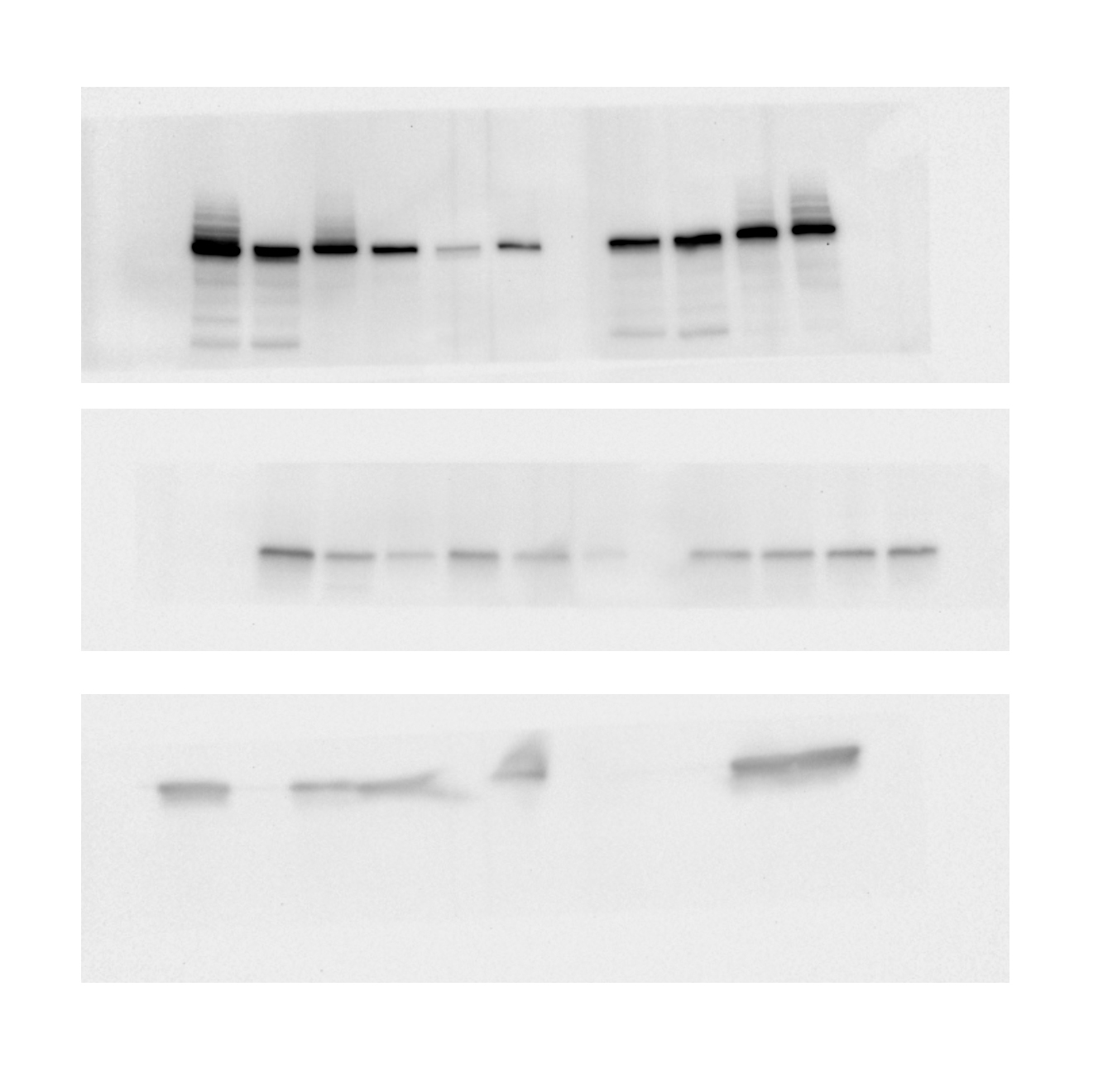

Supplement: Figure 8—source data 2. [file elife-95337-fig8-data2.zip › Figure 8-source data 2/Figure_8C-Original.jpg]

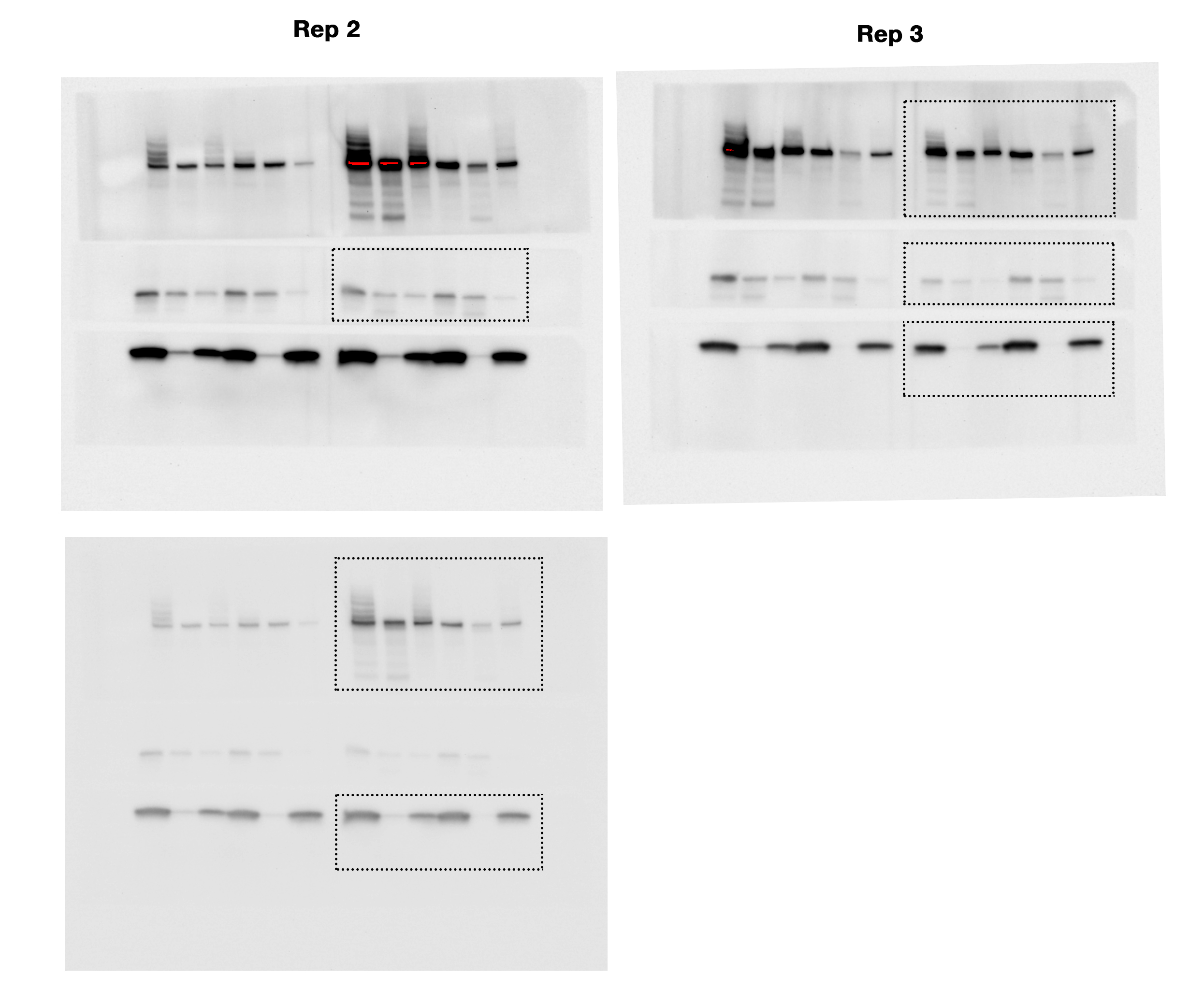

Supplement: Figure 8—figure supplement 2—source data 1. [file elife-95337-fig8-figsupp2-data1.zip › Figure 8-figure supplement 2-source data 1/Figure 8-figure supplement 2-Original-marked.jpg]

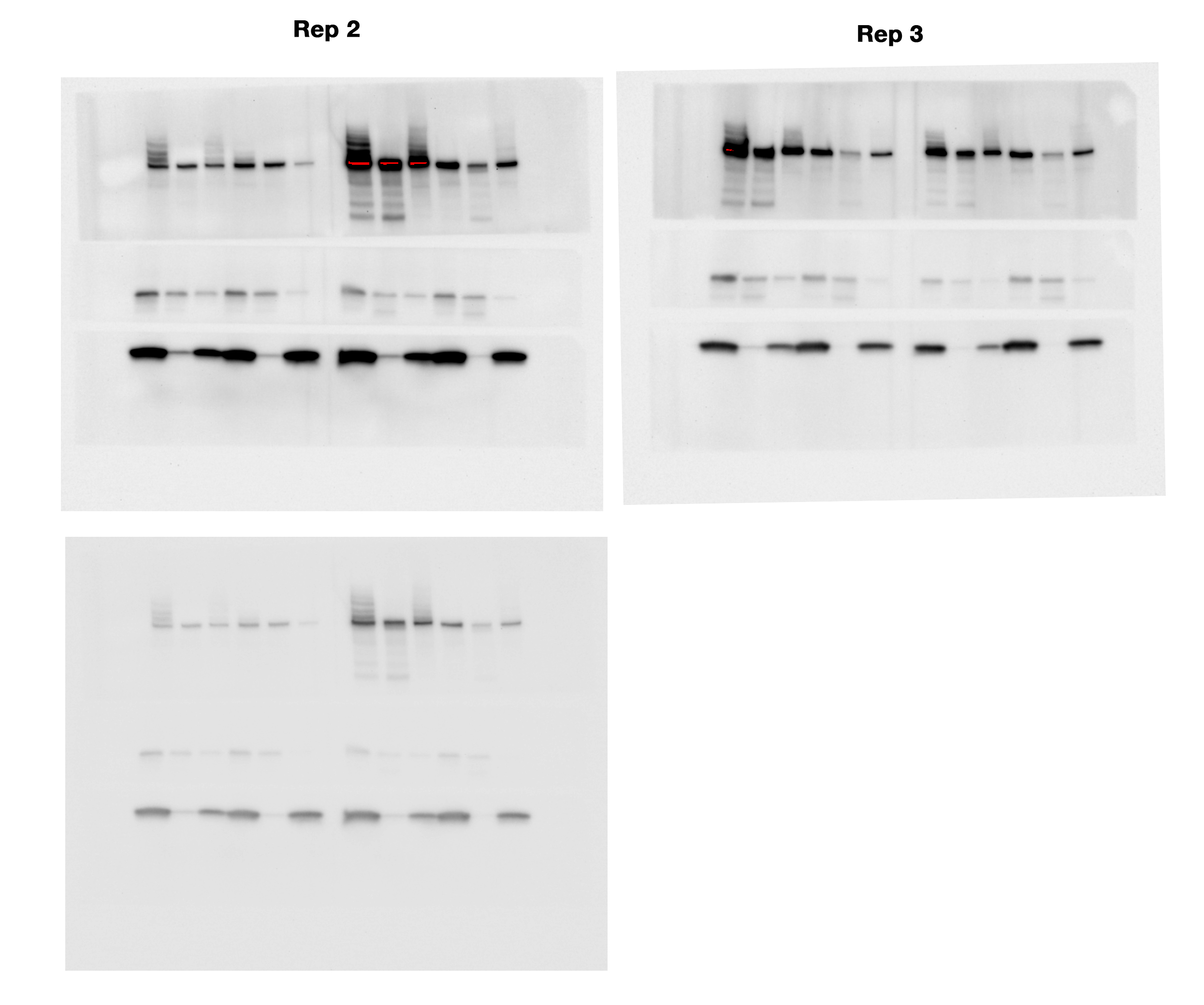

Supplement: Figure 8—figure supplement 2—source data 1. [file elife-95337-fig8-figsupp2-data1.zip › Figure 8-figure supplement 2-source data 1/Figure 8-figure supplement 2-Original.jpg]
