# Supplementary material for: A SUMO E3 ligase promotes long non-coding RNA transcription to regulate small RNA-directed DNA elimination
Source: eLife. 2024 Jan 10;13:e95337. doi: 10.7554/eLife.95337 (PMC10830130; doi:10.7554/eLife.95337)
Supplement: Supplementary file 1. [file elife-95337-supp1.docx]

## Supplementary Information

**Oligo DNAs**

EMA2-C-FW: CCGCTCTAGATTTAATCTTTCTTATGTTAATAATCGTC

EMA2-HA-C-RV: GTCGACACTAGTGGATCCAATGCAAATATTCAAAGAGTT

EMA2-F-FW: GGATCCACTAGTGTCGACTGTTTGTCTTTATCTCGTAGTC

EMA2-F-RW: GGCGCTCGAGTTCTAGGATAACTAATAGAGTGAAC

SPT6-3F-FW2-overlap :TGCTAGCGGATCCGTCGACCTCGAGTCTCTTAGTGTTTTATATAAAACAGC

SPT6-3F-RV1-KpnI:  ATTGGGTACCGGGCCCGTATCTTTAACTATCGACAAAACC

SPT6-FW1-SacII: TCCACCGCGGACTTATCACTTAAGGAAGAATATGGC

SPT6-RV2-overlap: ACTCGAGGTCGACGGATCCGCTAGCATGCTCGTTGTCATATGAACGAG

SMT3_HA_FW:CCTTATGATGTTCCTGATTATGCTGGTGGTTCTGGTACTGATTAAAACGC

SMT3_HISEXT_RV: GCTGACCGATTCAGTTCGCTCAATCAGAAAGAGCCACCAACTTGTTC

BTU1_LCFW: AATTTTAATGCGTGTATTTATTTGGGTG

MTT1_5UTR_SeqRV: AGTACTCTATTATGTTTTTTCTTATACAG

EMA2-pBNMB1-FW: ATGATGTTCCTGATTATGCTGGATCCATGAGTTCACTATTTGGAAATAAGAATAA

EMA2-pBNMB1-RV:TTAGCTGACCGATTCAGTTCGCTCAACTAGTTCAAATGCAAATATTCAAAGAGTTAAGAG

SPT6_FW_Turbo: GATCTGGTGGTTCAGGAAGCGGATCCATGAGTAAACCAAGAAAAAACGAAG

SPT6_RV_Turbo: GCTGACCGATTCAGTTCGCTCAATCAATGCTCGTTGTCATATGAACGAG

SPT6_N_FW_HA: GTATCCTTATGATGTTCCTGATTATGCTGGATCCATGAGTAAACCAAGAAAAAACGAAG

SPT6_N_RV: ATACTTTGCGCCATAATCACCTAC

SPT6_5F_FW: CAAATTTTTACTGGAAAAATGCAGCTAAATTATGAGACCAAACTAAG

SPT6_5F_RV_HA: GCATAATCAGGAACATCATAAGGATACATTTTTCAAATATTTGCTGTGAAGACT

SPT6-KO-5F-FW: CGAATTGGGTACCGGGCCCCCCCTCGAGCATAATATCATTCTCCACTCAC

SPT6-KO-5F-RV: CAGTAAAAATTTGGATCCTATCGAATTCCTGCAGCCCTGCTTAAAAGTGCCTATGTTAGC

SPT6-KO-3F-FW: CGATAGGATCCAAATTTTTACTGGAAAAATGCAGCCCTCTCGTTCATATGACAACGAG

SPT6-KO-3F-RV: GGCGGCCGCTCTAGAACTAGTGTATCTTTAACTATCGACAAAACC

SPT6_M249_FW: CTTATGATGTTCCTGATTATGCTGGATCCATGTAGGGACGTAAAGCTCCTGATCC

SPT6_M249_RV: GTTCGCTCAATCAATGCTCGTTGTCATATGAACGAGATCTAGAGCG

M5'-3: AGGTACGATAGATCGACTGACGG

M5'-4: AATAATAAGGAACCTCTTACTGTG

M3'-3: ACTAAAATATTTATCTTCTTTTCTGC

M3'-4: ACTTTCAAAAAATTAATTTTGAGTAAAG

L8_5'-1: ATTTGCTTTTAGGAAGAGATATCAC

L8_5'-2: ACATACATTTGAAATATCACCAAGC

L8_3’-1: ACAAGAAAGGTATTCATTCATTCCTC

L8_3’-2: TGATAAAAAAGTGGTGAAAAAAGGAC

R2_3'-1: TAATTTTAGGGCGAATCACC

R2_3'-2: ACTCATAAATAAAATCATATCATAGTCTAG

R2_5'-1: TTAGCAAAGTGCATTAACCTC

R2_5'-2: TCTCTTGAAATTGGGCAAAAACTG

RPL21-FW: AAGTTGGTTATCAACTGTTGCGTT

RPL21-RV: GGGTCTTTCAAGGACGACGTA

NMC1_f53600: GTGCAATTTTACACCGTCAGG

NMC1_r55732: AGAATCTAACTCATAGCACTGG

**Synthetic DNAs**

>EMA2-Ec

CGATCGGGATCCATGTCTAGTTTGTTCGGTAACAAGAACAAGGAAGTTATCTTCAATTTTTGCCAAAAGTCTCAGGAAGAAGATCAAGACATGTTTGCGGACCATGAAATTCTGATTGATGAACAATTACAGTGTTTTAACTGTAACAAGTACTTTGATCCCACGAAGTACGGGATCGATTTGTTAGACGAGATGGACTTCAACAAGAATCTGGACACTTTTTGTTGTGAGAATTGTTATCTTTCGTTGCATTTTCCTTTTTATTATCAATTGGATTTATTGTTGAAAATGTCAAACCTGGAAGTGAACACGAAGTACGAATTCAACCTTGTACTGCAGGAGGAGTGCTACCAAGACAGTTGCATCCTGGGCGTGTTCTTAAGCAAGCAAAATCTGATTAATGTCTTCAAGAAACAGACGGGGAGTCATAACTTCATCTTAAACGTGAAAATGAATGGGCAAGACCCCTCCCAGGAAGGCAGTAACTTTTTTGTGATCGACTCGGATGACTTAACGCAGATGCAAAATACTTTGAACTTCGAATGTCTTAACTTTTCCTTTCAGAAATCTTTCATCTCGAACATCATCTTAATGAACGACCAACAGAAAAGTCAGCTTAAAACGGCCTCTCCGAACATCATCAGCGTAATCCTGCACTGCCGCAAAATTCGTGTCTCTGCGTATGTCGCGAAACTGTTTAAGAAGAAGTTCTTTAACTGTTTAAATCATGAAAGTTTATTCAAGGTTTTCGAGGAACAAGAGCAAAAATATAAGCAAAAAGACCAGATCCAGAAAATCAAATTACAAACCAAACAGAACTCGCTTTCAACGTCTCAAATTCATCAGTCTAGTACCCTGAACGAAATTCAGCCCAGCCAGCTTCAAACCCATAAGGAAACTTCGAACATCTCGCAAAGTAGCCAGCTTAACAAAGATTTCTGTGAAAAAAAAGAACTGACCGAGATCTATCTTCGCATTAAAAACTTTCACCAGATGTTCCCGTTCATGTACAAGAATGAGATGGTGGTTAAGATGGACGATAACGCAGTTATCCAATACCCCGCGTTTACCTACCATCACTCGCACCCCTTTGACTTACGTGATTACTGCTACCTTAACCAGGTGAATCCTACCTGGTTGTGTCCCATCTGCAACAAGCACAAGATTTTCTTGAAGGAGATCCAGCTGGATTTTTATTTGTTTGCGTTGATTCAAACACAGTATCTTAAGGACTCCTATATTTCGGACAAAATCCAGCAGATTCTTCAGTCGAAGCCACAGTTTGATTCTGAATTTTTTAGCAATCAACTGAAACAGCTGAACTTGCACTCAATGAAGGTACTGGAGCAAACACAGCAACGCACCCAGTCTAACGAGATTAAGATCCAGCAGAACTTATCCAATAAGTTGAACCAGACTAACAAGAATTTTATCCAGATCTACAAAAAGTCGAATCAGTTGGGGCAAAAAGAGAATATTCTGAAGAACAGTACTAAAATTAAGCAATTTAAGTGTAAGTCATCTATTGATCAGAATACAAATTATGAACAGCGTAACCACAAGAATGGGAATCAAAATCAAAAGTCAAAAACCTCAGAGTTTTTCAATGAAAATTTGAACTGTACTGACTTAAGCCAAGACAATTACCTGGATTTCATTCAGGATTTTGACGAATTTCAATCTTTCTTAAATCCTTACCAGAAGCAACATTCGATGCGCAATAGCAAAGATATTTCTGTACGTCATAATATGGATCAAGCTAACCTTATCGACGAGATGTCTATCCTTAACATTCAGTGCAATGAAATCGCCTCCCAAAAATCTTCAACCCGTGTTTACAGCAAATTAGTAAACAAATCTAAATCGAAGACCTCATCTTCTAACAAAGAAGAGAAGCAGAAGCAGAACGACGCATTTAAAGAAAACCAAATCGACATTGAGCAATCAGGCCAGATTATCGAAGAAAAAGTCCAAGAGAAACCGAGCTTGAACGATCAAATTGATGCTAATAAGAACATCCAGTTCTCCCAACAAATTGAGTCCTTTTCCGACCTGAGTCAGAAGTTCATCTCCCAGCAAAAAGAAGAGCGTACTCTTGAGGTTGCAAATCAATTCTACTTGGACACTTCGTTCCTTCGCTCCCCTCTTGAAATCAACGTCTTGTTTGACGTATCCTATTTGCAATTCAATAAAGAAGAGCAGGAGTATCAGTCAGTTCAGCAGATCATTCAGCTGATTGACTTCCAAGAAGACAAGCAGATGTTAGAGAAGCTTTTCAACATTGACATCCTTCAAAATCACTATTTTATTTTCAATATTTTCAATCTGAGCTATGTAAATAATCGTCAGTATCAAGACTTGCACACAATCTTAAACCAAAACCCTTCGACTAAAACTTTTTTTCAAGTCTTGATCAACGTTCAAGAGCAGTTCATCAACCTTGGTATGAGTTCAGAGTTTTATACGTTAATTTCTCAGTTCTATTATAAAGTTATGTCTAATAAGCAGCGCAATAATAAGATTTCTTACGACCTTTTGGATTCCGAAAGCCAAATTACGTCGCTTAATTATTCGCAAAACAAAAACTTTTTAGAGAAACTTATTGAATGCTTCCTGGGAATGTCGATTTTTAAGCAACTGTCCAACGAAGATGTCATTGAAAATTTCCAACAAATGGTCTTTGAATACTTATTGTTCAAGCAACAAAATATCCCGACTTTGATTAATATTCACAATATCATCATCAACTATTTTCCCAATCAATGCCTTAACTTAATCGAATCGATCAAGAAATCTTTGAATAGCCTGAACATTTGCATCTGACTCGAGCCCGGG

*>*UBC9-Ec GGCCTGGTGCCGCGCGGCAGCCATATGATGCAACAGCAGAATAAAGAAGTTAACGAACTTGTAATTACGCGCCTGAAACAGGAGCGCAAACAGTGGCGTCAGGATCATCCTCATCGTTTCGTCGCCAAACCCATGACTAAAGAAGACGGTACCATCAATATGCTTAAATGGTACTGTGAGATTCCGGGACCGGAAGGGTCACCCTGGGAGGGGGGCGTTTACATTTTATACATGGACTTTTCACAAAGCTACCCTATTAAACCCCCTAAGTGCCAATTCAAACCGGTCCTTCCTCACCCCAACGTGTATCCAAGTGGCACCGTCTGCTTATCAATCCTGAACGAAGAAGAGGACTGGAAATCTGCAATTACGATCAAACAGATTTTGATGGGAATCCAAAAGTTACTGAAGGATGAACCGAACATTGACAGCCCTGCCCAGTACGAGCCCTGTGCTTTATATCGTAGTGACAAGGAAAAATACTACAAAAAGGTACGTGAGTTTGCTGAAACTATGAAGAAGAAGGATtgagGATCCGAATTCGAGCTCCGTCGACA

>SPT6-DOL-KtoR

CCTTATGATGTTCCTGATTATGCTGGATCCATGTCTAGACCTCGTCGTAACGAGGAGGTTGATGATCAGATGAGAAACATCGGAGATGAAGAAGAAGAAGATTATCAAGGACAGGTCGAGGAAGAAGAAGATGGAAACAATCAAGTCGAGGAAGACGACGAGGAAGAAGAGGATGATGACGAACAAGACGATTACCAACAGGACGGATTTGTCGTCGGAGATAGCGAAGAAGAGGAGATAGAAGAGGAAGAAGATGAAGATGCCGAAGAGAGACGTCGTCGTAGAAGAGAACGTAGAAGACAAAGAAGATTGGAGAGACAAAACCAGCACAGAAGAATACTCAGACGTGGTCCAAGACGTCGTGAGTTGAGCGAGGATGAAATGGAGAACAATGAGATTCAAGATTTAGAAGAGTATCATGAAGACTCTGAAGAGGGTATTCCTGACCGTCGTAGAAACCAGCAGTCACGTACAGGAAACGACGGTCACCAGGGAGACAGAATGGACCTTGAGGACCAAGGTAATGAGGACTATCTTGAGGACGTTGGTGACTATGGAGCTAGATACGGAAGAACAGCCTTCGAAGAACTCTTTATGGATGATGAGTCAGGTGAAGAGGAACAGAACGAGAATGAAGACGACGAGAACCGTGAAGTAGACGACTACATCGACGTCCAACAGTTGTTCGAACCCGATGACTTGCGTAGACGTTTCGAAAGAGACGACGACCGTAGAATTCGTGAAGAGGATATACCCGAGCGTCTCCAAATCCGCATGTAGGGACGTAAAGCTCCTGATCC

>SPT6-118KR-gBlock-1

CCTGATAGAAAGAGAAATCAGTAAAGTAGAACCGGTAATGATGGTCATTAAGGAGACAAGATGGATTTAGAAGATTAAGGTAATGAAGATTACTTAGAAGATGTAGGTGATTATGGCGCAAAGTATGGAAAAACAGCCTTTGAAGAACTTTTCATGGACGATGAGTCAGGAGAAGAAGAGCAAAATGAAAACGAAGATGATGAAAATAGAGAAGTTGACGATTACATTGATGTGTAATAGCTTTTTGAACCTGATGATCTTAGAAAGCGTTTCGAGCGTGATGATGATAAAAAAATTAGAGAAGAGGATATACCTGAGCGCTTATAGATTCGTATGTAGGGACGTAGAGCTCCTGATCCAGAAGAGCTCATGGAAGAGACAAGATGGATATCAGAAAAAATTAGGACTATAAGGGATTATAGGGGTAGCAGGAGGTTGAATGATATTAATTTCCATTCAAAAATATTCTCTTTCCTCCAATCTCTACATTTGGATAAAGAAGAAGTTATGTATATCTATACATACAAGAGGAATGAATTTCATCCACAGTTTGATCTTGAAGACTTATGGAGATTATATGACTTAGATGGCGAATGGGCTCAATTCAATAGACAAAAAAATCGCATTCTATTATAAATTTAATAGCTTAGAAGAGAACTTGAGAATACTCCTCTGCTAATTTAAAATAATATAATTGATTTTGAATAAGTTAGAAGAGTTGAAGAATTTTTCCACAGAGCCATTGATACTTAATCTCTTAAGTTTATCAAGGAATACTTTGATTACCTATTTATCAAAATTTATCCTTAAAGGGAGCACTAAAGATTAAGAATTCGTAGAGACCGTAAAACTAGAATGATTAACACTTATATTTAATGTAGGGTTCATAGGTTAGTTAGTACTTTAACTCTTTCTCCTTAGGATCTCGTTAGAAACTTAGAAGAAATGAACTAAATTAATTAACCTCAATTACAACAAAATAGGCCCTAAGCCTATGCTGATAATTTAATTTCAGAAAACCCAAATGTTCCTTGTATGAGAGAAGCTATAGAGGCTTTAGAATGCATGGTTGATTATCTATCAAATGAATTATTTAACTATCCTCCCCTTAAAAGATGGTATTATGAAATGTTTAGATAAAGATGCTTAATTTCTACTGAACCCACAGATCTTGGTAGAAGGTAAATAGATATGTTCCATCCCCTATACCCAGCTAAAAGAATTCATAGACGTCCTTACACTTCTTTCACTGATGACACTTGGTTGCTCTTAGAAGAAGCTGAAAGATTGAACTTTATTACAGTTAGGATTTTCTTAAGAAGTGAAAATGATAATAACTCAGACAATAACAGCATATTTGATATTATGAGTAGAAATTACATGATCAGTTTAAGAAATTTAGAAGCTCAAATTAGACAAATTAATTCTGAATGGAATTTCTTCCGTAGATACAACATAGAAAATGTTTGTGATAGGTTTTTCTTACCT

>SPT6-118KR-gBlock-2

CATAGAAAATGTTTGTGATAGGTTTTTCTTACCTTATGCCTAGAGCTTAGTAAGAATAGAACTTCATGAAAATGCTGAAAAGTGGGTTGTATCTTAATGCTAACAAAGGTTTTACAACATGATTAATGTTGAACCTTAGAGAAATGGTAGAATAATGTCAGTTGTCCAAGACACAAATGGTAGAATAGGTGTTGTCTTTGTTGATGAAAATGGAATTCCTAATAATTCTATGATTTTGAACTACTTCACCAGGAGAGAAGACTAGCTTTCAACTAGAGCCAGACTAGAAAAAACCTAAGAAGAACATGAACTTGAAATTAGGTTTAGATCTTTTGGACCTTAATTAATTGTTGTTGCTGCCAATAGCATAGAATCTTAAAGACTTAGAAATCTACTTCATTAAAAATATGAAAGATCTTAATCTTATATTTAATATGGAGATGACACAATCCCATAAATTGTGGCTAAGTAACCTACAATTCAAGGACCAGGAAATACCTTACAATTTAGAGATTCTGTACTCAGAGTTGCTCTTTCATAAGGAAGAATACTTTTAAATCCTACTGCAGAAATACTCTCGCTTTGGAACGATAATGTTTAACAAAACGGGTGTCTTCATATCCCCCTTCATCCATCTTAAGAATATATTAGTCTTGATAAGCTCTAGTACTCACTAGAGAAATGTTGTGTTTAAATAGTCAATTTTATGGGTGTTGAAATGGATATGCTTTAAGATTAGCCTCACCTTAGACACCTTCTATAACACGTATGTGGTTTAGGACCTAGAAAGGCATTAAGGCTAATTGAGCTCTTAGAAAGAAATGCTGTAATTTTTGAAAGAGAAAGGGAAGTACCTAAAAGGAGAGGTCTATTAATTACTCAACTTGATATAAGGGATGTTGTATTCAGGAATATTAATGGTTTCATCAGATTTAGATTTTCAAAAGACCCTATTGAAAAGACTAGAATCAACAGAGATTTATACAGAATTGCAAGAAGGATTTGCAGAGACAGTGCTGATAACTTCTAAATTAGAAGCTAAAGTGATGAAGATATAGTAGAATATGTTATGAGAAATCCCAGATATATTGATGCTATGGACTTGGAAGATTATGCTTAACAGCTTGAGGAGATGAGAAATCAACCAAACATGGCTCCTGTATTAGATTTTGTCAGAGATGAGCTTATCAACCCATTTAGCTATAGAAGAAATACCTATGAAGCAATTAGCGATTAAGACTTATTCTTCAAAATGATTAGAGAAAGTCCTCAAACATTCCGTAAAGGAATGATTGTTTCAGCAAGAATCATTCAAATTAGACCTAGAGAAAATCAATAAATAAGACAACACTAACTACTTGTTAGGATTGTTGATAACGACCTAAGATCTAGCATACTTGTTAGTGAAGAGGAATCAAGGAATTATAGGGTTGGTGATATTATTAAAGCTTATGTTGATCAGATTTTTGTCCTTGACAACAGGCGC

>SPT6-118KR-gBlock-3

GATTTTTGTCCTTGACAACAGGCGCTCCAAGGATAGAATTATTGGTTTCGATGTCAATTGTGTGACTTTAACTTTTAGAGCCATCAGATTTAATGATTTAGTCAGAGAAATGAGATAATTTTAAGACTTAGATATTCTTAGTACTTTCAGGTTCATTGAAGCTGAAGACAGGCCATTGGGTATTGATGTCACTGAAAGAAGAGCTAGGAGATTCGAACCAAGAAGGATTGCTCATCCTAATTTTAGAAATATAAGTATTACAGATGCTGTAAGGCTTTTGAGGAATGCAAGAAATGGTGAATTTATTATTAGACCCAGTTCAAAAGGTAGACAATACCTTGCCATAACATGGAGATTCTTCGATGACGTGTTTGTCCACTTATCACTTAGGGAAGAATATGGCAGAGAAAGGGGGTTCTAAACGAGATATGTTTTAAATGACAGAGAATCGTTTGATAACTTTGATGAAATTATTGAAAGATATATCATTCCTTGCAATAATCACATGAACTCAGCTAGAGATAATCGTAGGTTTTCAAGAAGATCAATAGAAGAAATAGAATAGGAGCTAAGGAGAAATAGAGAAGAATAACCAGACATTATTCACTATAACTTTTGTTGTGTCCCTAGATATCCTCAATTTATCGTCTTGCTATATTGCTCTAGATAGGATTAAGTCACCAAAGAGTGGATTAAAGTTAAGTATTAGGGCTTTTATTTCCATGAAAGGTATTTTGGACAATTAAGGGATTTAATTAGATGGTTTAGAGATTGCTTCCATACTCCCGAATACAGAAGGTATGTAAAAAGAGCTGAAGAACCATATGCATCTAGGACCCCTAGCTCATTTAGTAATTAAGGAACTATTGGCATAAGATAAGAAGGTGGTGTCAGATATGAAAGATCAGAGAGAGGCATTGGAGGACAAAGATGTTAAAACTGTGGTAGATATGGTCATGCTGCTAATAACTGCAGAAATAGAAGATCCTATAATTCCTCTTCAGGATAGGGCAGTGCATCTGGAGCAAAACAATGTTTCCACTGTAGGGGTACTGACCACTTTATTAGAGACTGCCCAAATAGAACTCATCAAAGCAATAGAGGAAGTGGCGGTTTTAGCTATAGCAGAAGAGATAGAAATCAATAACACTCACGCTCTAGATCTCGTTCATATGACAACGAGCATTGATTGAGCGAAC

>SPT6-42KR-gBlock-1

CCTGATAGAAAGAGAAATCAGTAAAGTAGAACCGGTAATGATGGTCATTAAGGTGATAAGATGGATTTAGAAGATTAAGGTAATGAGGATTACTTAGAGGATGTAGGTGATTATGGCGCAAAGTATGGAAAAACAGCCTTTGAGGAGCTTTTCATGGACGATGAGTCAGGAGAGGAGGAGCAAAATGAAAACGAAGATGATGAGAATAGAGAGGTTGATGATTACATTGATGTGTAATAGCTTTTTGAGCCTGATGATCTTAGAAAGCGTTTCGAGCGTGATGATGATAAAAAAATTAGAGAAGAGGATATACCTGAGCGCTTATAGATTCGTATGTAGGGACGTAAAGCTCCTGATCCAGAGGAGCTCATGGAGGAGACAAGATGGATATCAGAGAAAATTAGGACTATAAGGGATTATAGGGGTAGCAGGAAGTTGAATGATATTAATTTCCATTCAAAAATATTCTCTTTCCTCCAATCTCTACATTTGGATAAAGAAGAGGTTATGTATATCTATACATACAAGAGGAATGAGTTTCATCCACAATTTGATCTTGAGGACTTATGGAGATTATATGACTTAGATGGCGAATGGGCTCAATTCAATAGACAAAAAAATCGCATTCTATTATAAATTTAATAGCTTAGAAGAGAGCTTGAGAATACTCCTCTGCTAATTTAAAACAACATAATTGATTTTGAGTAAGTTAGAAAAGTTGAGGAGTTTTTCCACAAAGCCATTGATACTTAATCTCTTAAGTTTATCAAGGAGTACTTTGATTACCTATTTATCAAAATTTATCCTTAAAAGGAGCACTAAAGATTAAAAATTCGTAAAGACCGTAAAACTAGAATGATTAACACTTATATTTAATGTAAGGTTCATAAGTTAGTTAGTACTTTAACTCTTTCTCCTTAGGATCTCGTTAGAAACTTAGAAGAGATGAACTAAATTAATTAACCTCAATTACAACAAAATAAGCCCTAAGCCTATGCTGATAATTTAATTTCAGAAAACCCAAATGTTCCTTGTATGAAAGAAGCTATAGAGGCTTTAGAATGCATGGTTGATTATCTATCAAATGAATTATTTAACTATCCTCCCCTTAAAAAATGGTATTATGAAATGTTTAAATAAAGATGCTTAATTTCTACTGAACCCACAGATCTTGGTAAAAAGTAAATAGATATGTTCCATCCCCTATACCCAGCTAAAAGAATTCATAAACGTCCTTACACTTCTTTCACTGATGACACTTGGTTGCTCTTAGAAGAAGCTGAAAGATTGAACTTTATTACAGTTAGGATTTTCTTAAGAAGTGAAAATGATAATAACTCAGACAATAACAGCATATTTGATATTATGAGTAAAAATTACATGATCAGTTTAAAGAATTTAGAAGCTCAAATTAGACAAATTAACTCTGAATGGAACTTCTTCCGTAAGTACAACATAGAAAATGTTTGTGATAAGTTTTTCTTACCT

>SPT6-42KR-gBlock-2

CATAGAAAATGTTTGTGATAAGTTTTTCTTACCTTATGCCTAGAGCTTAGTAAGAATAGAACTTCATGAAAATGCTGAAAAGTGGGTTGTATCTTAATGCTAACAAAAGTTTTACAACATGATTAATGTTGAACCTTAGAAAAATGGTAAAATAATGTCAGTTGTCCAAGACACAAATGGTAGAATAGGTGTTGTCTTTGTTGATGAAAATGGAATTCCTAATAATTCTATGATTTTGAACTACTTCACCAGGAGAGAAGACTAGCTTTCAACTAAAGCCAGACTAGAAAAAACCTAAGAAGAACATGAACTTGAAATTAGGTTTAAATCTTTTGGACCTTAATTAATTGTTGTTGCTGCCAATAGCATAGAATCTTAAAGACTTAGAAATCTACTTCATTAAAAATATGAAAAATCTTAATCTTATATTTAATATGGAGATGACACAATCCCATAAATTGTGGCTAAGTAACCTACAATTCAAGGACCAGGAAATACCTTACAATTTAAAGATTCTGTACTCAGAGTTGCTCTTTCATAAGGAAGAATACTTTTAAATCCTACTGCAGAAATACTCTCGCTTTGGAACGATAATGTTTAACAAAACGGGTGTCTTCATATCCCCCTTCATCCATCTTAAGAATATATTAGTCTTGATAAGCTCTAGTACTCACTAGAGAAATGTTGTGTTTAAATAGTCAATTTTATGGGTGTTGAAATGGATATGCTTTAAGATTAGCCTCACCTTAGACACCTTCTATAACACGTATGTGGTTTAGGACCTAGAAAGGCATTAAGGCTAATTGAGCTCTTAGAAAAAAATGCTGTAATTTTTGAAAGAGAAAAGGAAGTACCTAAAAGGAGAGGTCTATTAATTACTCAACTTGATATAAAGGATGTTGTATTCAAGAATATTAATGGTTTCATCAGATTTAGATTTTCAAAAGACCCTATTGAAAAGACTAGAATCAACAAAGATTTATACAAAATTGCAAGAAAGATTTGCAGAGACAGTGCTGATAACTTCTAAATTAGAAGCTAAAGTGATGAAGATATAGTAGAATATGTTATGAAAAATCCCAAATATATTGATGCTATGGACTTGGAAGATTATGCTTAACAGCTTGAGGAGATGAAAAATCAACCAAACATGGCTCCTGTATTAGATTTTGTCAGAGATGAGCTTATCAACCCATTTAGCTATAAAAGAAATACCTATGAAGCAATTAGCGATTAAGACTTATTCTTCAAAATGATTAGAGAAAGTCCTCAAACATTCCGTAAAGGAATGATTGTTTCAGCAAAAATCATTCAAATTAGACCTAGAGAAAATCAATAAATAAGACAACACTAACTACTTGTTAGGATTGTTGATAACGACCTAAGATCTAGCATACTTGTTAGTGAAGAGGAATCAAAGAATTATAAGGTTGGTGATATTATTAAAGCTTATGTTGATCAAATTTTTGTCCTTGACAACAAGCGC

>SPT6-42KR-gBlock-3

GATTTTTGTCCTTGACAACAAGCGCTCCAAGGATAAAATTATTGGTTTCGATGTCAATTGTGTGACTTTAACTTTTAAAGCCATCAAATTTAATGATTTAGTCAGAGAAATGAGATAATTTTAAGACTTAGATATTCTTAGTACTTTCAAGTTCATTGAAGCTGAAGACAGGCCATTGGGTATTGATGTCACTGAAAGAAGAGCTAGGAGATTCGAACCAAGAAAGATTGCTCATCCTAATTTTAAAAATATAAGTATTACAGATGCTGTAAGGCTTTTGAAGAATGCAAAAAATGGTGAATTTATTATTAGACCCAGTTCAAAAGGTAAACAATACCTTGCCATAACATGGAAATTCTTCGATGACGTGTTTGTCCACTTATCACTTAGGGAAGAATATGGCAAAGAAAAGGGGTTCTAAACGAAATATGTTTTAAATGACAAAGAATCGTTTGATAACTTTGATGAAATTATTGAAAGATATATCATTCCTTGCAATAATCACATGAACTCAGCTAAAGATAATCGTAAGTTTTCAAAAAAATCAATAGAAGAAATAGAATAGGAGCTAAGGAGAAATAGAGAAGAATAACCAGACATTATTCACTATAACTTTTGTTGTGTCCCTAAATATCCTCAATTTATCGTCTTGCTATATTGCTCTAAATAGGATTAAGTCACCAAAGAGTGGATTAAAGTTAAGTATTAGGGCTTTTATTTCCATGAAAAGTATTTTGGACAATTAAAGGATTTAATTAAATGGTTTAAAGATTGCTTCCATACTCCCGAATACAAAAAGTATGTAAAAAGAGCTGAAGAACCATATGCATCTAGGACCCCTAGCTCATTTAGTAATTAAGGAACTATTGGCATAAGATAAGAAGGTGGTGTCAGATATGAAAGATCAGAGAGAGGCATTGGAGGACAAAAATGTTAAAACTGTGGTAAATATGGTCATGCTGCTAATAACTGCAAAAATAAAAGATCCTATAATTCCTCTTCAGGATAGGGCAGTGCATCTGGAGCAAAACAATGTTTCCACTGTAAGGGTACTGACCACTTTATTAAAGACTGCCCAAATAAAACTCATCAAAGCAATAAAGGAAGTGGCGGTTTTAGCTATAGCAGAAGAGATAGAAATCAATAACACTCACGCTCTAGATCTCGTTCATATGACAACGAGCATTGATTGAGCGAAC
